# Supplementary material for: Early Emergency Medicine Milestone Assessment for Predicting First-Year Resident Performance
Source: MedEdPORTAL. 2024 Mar 12;20:11386. doi: 10.15766/mep_2374-8265.11386 (PMC10928014; doi:10.15766/mep_2374-8265.11386)
Supplement: Supplementary file 1 — MED Stations and Schedule.docxSample EM PGY 1 Orientation Didactic Syllabus.docxMED Checklists.docxMED Station 1 Materials.docxMED Station 2 Materials.docxMED Station 3 Materials.docxMED Station 4 Materials.docxMED Station 5 Materials.docxMED Station 6 Materials.docxMED Station 7 Materials.docxMED Performance Summary.docx [file mep_2374-8265.11386-s001.zip › A. MED Stations and Schedule.docx]

| **Appendix A. Milestone Evaluation Day Stations**  **Instructions for Facilitators: Each station below includes station name, time allotted, and setup required. Please review these guidelines in preparation for MED.** | | | | | |
| --- | --- | --- | --- | --- | --- |
| **Station #1A & #1B** | **Time (minutes)** | **Milestones Assessed** | | | **Personnel Needed** |
| 1A. History/Physical Exam | 5 | PC2, PC5, PROF1, ICS1, SBP3 | | | 1- actor/evaluator |
| 1B. Presentation & Differential Diagnosis | 5 | PC2, PC3, PC4, ICS2 | | | 1- evaluator |
| Setup 1A: Classroom 1/faculty office – Empty stretcher for actual actor, chair for learner next to stretcher. Notepad.  Setup 1B: Classroom 2/faculty office – Chair set up near computer for learner to present to evaluator/attending. Notepad. | | | | | |
| Details 1A: Faculty will be a scripted patient. Following the encounter, they will assess the learner’s history and physical exam. Checklist provided.  Details 1B: Learner will present patient to evaluator in adjacent classroom. Evaluator will mark off that learner is able to present all data concisely and in an organized fashion. Checklist provided. | | | | | |
|  | | | | | |
| **Station #2** | **Time (minutes)** | **Milestones Assessed** | | | **Personnel Needed** |
| Patient Simulation | 10 | PC1, PC2, PC5, PC6, PC7, PC8, ICS1, SBP3, PROF1 | | | 3- nurse role-player, evaluator, faculty to run Laerdal SimMan |
| Setup: Simulation room – Empty stretcher for Laerdal SimMan, EM cart, defibrillator, regular gloves, chair and bedside table for evaluator next to stretcher. | | | | | |
| Details: One faculty will run a simulated case and another will roleplay the nurse. The simulation will be of a patient who goes into SVT. The learner must go through the full patient scenario, make the correct diagnosis and give the correct treatment. The evaluator will fill out the checklist provided. | | | | | |
|  | | | | | |
| **Station #3** | **Time (minutes)** | **Milestones Assessed** | | | **Personnel Needed** |
| Venous Vascular Access | 5 | PC9, PC14 | | | 1- evaluator |
| Setup: Bioskills/simulation area – Peripheral IV/venipuncture trainer, skin prep, IV supplies (18g if available), regular gloves, sharps container. | | | | | |
| Details: Evaluator will observe resident prep skin and successfully perform venipuncture. Check list provided. | | | | | |
|  | | | | | |
| **Station #4** | **Time (minutes)** | **Milestones Assessed** | | | **Personnel Needed** |
| Wound Management | 10 | PC9, PC11, PC13 | | | 1- evaluator |
| Setup: Bioskills/simulation area – Wound Care station with pork belly, betadine (or other sterile prep), several suture types to choose from (4.0 and 5.0 absorbable and nonabsorbable), lidocaine, sterile gloves, lac tray kits, sharps container. | | | | | |
| Details: Evaluator will observe learner sterilely prep/drape pig skin, inject lidocaine through wound edges and place 3 simple interrupted sutures. Check list provided. | | | | | |
|  |  |  | | |  |
| **Station #5** | **Time (minutes)** | **Milestones Assessed** | | | **Personnel Needed** |
| Arterial Puncture | 5 | PC9, PC14 | | | 1- evaluator |
| Setup: Simulation area – Arterial line trainer, radial art line kits, sterile prep, sterile towels, sterile gloves, sharps container. | | | | | |
| Details: Evaluator will observe learners prep and drape arterial line trainer and successfully cannulate the artery. Check list provided. | | | | | |
|  |  |  | |  | |
| **Station #6** | **Time (minutes)** | **Milestone**  **Assessed** | | **Personnel Needed** | |
| Airway | 10 | PC9, PC10 | | 1- evaluator | |
| Setup: Simulation area – Airway station with mannequin that allows for jaw thrust/chin lift and intubation, adult mac/miller blades (3.0 and 4.0) with handle, adult nasopharyngeal airway, adult oral airway, bag-valve mask, 7.0 endotracheal tube with stylet, 10cc syringe, regular gloves, mask with face shields, sim lubricant for airway placement. Ideally a separate table for airway photo identification test. | | | | | |
| Details: Evaluator will ask basic airway landmark questions, and have the learner prove that they can successfully perform the following: 1. Appropriately hold and bag using bag-valve mask 2. Place a nasopharyngeal airway 3. Perform a chin lift and jaw thrust 4. Put together and hold a laryngoscope handle/blade (they may attempt to intubate if time permits). Airway photo, answers, checklist provided. | | | | | |
|  | | | | | |
| **Station #7** | **Time (minutes)** | **Milestones Assessed** | **Personnel Needed** | | |
| Written Test | 30 | PC5, PC11, PC12, MK, PBLI | 1- moderator/scorer | | |
| Setup: Classroom – Chairs and desk, pencils, written exam. | | | | | |
| Details: Moderator will provide learners with a written exam and score completed exams from the provided key. Passing is 85%. | | | | | |
| Abbreviations: PC, patient care; PROF, professionalism; ICS, interpersonal communication and skills; SBP, system-based practice; MK, medical knowledge | | | | | |

Example Schedule

| TIME | Station #1A & #1B | Station #2 | Station #3 & #5 (switch after 5 minutes) | Station #4 | Station #6 | Break | Break | Station #7 |
| --- | --- | --- | --- | --- | --- | --- | --- | --- |
| 9:00-9:15 | 1 | 2 | 3 | 4 | 5 | 6 | 7 |  |
| 9:15-9:30 | 7 | 1 | 2 | 3 | 4 | 5 | 6 |  |
| 9:30-9:45 | 6 | 7 | 1 | 2 | 3 | 4 | 5 |  |
| 9:45-10:00 | 5 | 6 | 7 | 1 | 2 | 3 | 4 |  |
| 10:00-10:15 | 4 | 5 | 6 | 7 | 1 | 2 | 3 |  |
| 10:15-10:30 | 3 | 4 | 5 | 6 | 7 | 1 | 2 | 8-14 |
| 10:30-10:45 | 2 | 3 | 4 | 5 | 6 | 7 | 1 | 8-14 |
| 10:45-11:00 | 8 | 9 | 10 | 11 | 12 | 13 | 14 | 1-7 |
| 11:00-11:15 | 14 | 8 | 9 | 10 | 11 | 12 | 13 | 1-7 |
| 11:15-11:30 | 13 | 14 | 8 | 9 | 10 | 11 | 12 |  |
| 11:30-11:45 | 12 | 13 | 14 | 8 | 9 | 10 | 11 |  |
| 11:45-12:00 | 11 | 12 | 13 | 14 | 8 | 9 | 10 |  |
| 12:15-12:30 | 10 | 11 | 12 | 13 | 14 | 8 | 9 |  |
| 12:30-12:45 | 9 | 10 | 11 | 12 | 13 | 14 | 8 |  |

Stations 15 minutes in length to allow for transition time

Resident 1 – 9:00-11:15

Resident 2 – 9:00-11:15

Resident 3 – 9:00-11:15

Resident 4 – 9:00-11:15

Resident 5 – 9:00-11:15

Resident 6 – 9:00-11:15

Resident 7 – 9:00-11:15

Resident 8 – 10:15-12:45

Resident 9 – 10:15-12:45

Resident 10 – 10:15-12:45

Resident 11 – 10:15-12:45

Resident 12 – 10:15-12:45

Resident 13 – 10:15-12:45

Resident 14 – 10:15-12:45
