# Supplementary material for: Early Emergency Medicine Milestone Assessment for Predicting First-Year Resident Performance
Source: MedEdPORTAL. 2024 Mar 12;20:11386. doi: 10.15766/mep_2374-8265.11386 (PMC10928014; doi:10.15766/mep_2374-8265.11386)
Supplement: Supplementary file 1 — MED Stations and Schedule.docxSample EM PGY 1 Orientation Didactic Syllabus.docxMED Checklists.docxMED Station 1 Materials.docxMED Station 2 Materials.docxMED Station 3 Materials.docxMED Station 4 Materials.docxMED Station 5 Materials.docxMED Station 6 Materials.docxMED Station 7 Materials.docxMED Performance Summary.docx [file mep_2374-8265.11386-s001.zip › C. MED Checklists.docx]

**Station #1A Checklist – History & Physical Exam**

Resident: _________________________________ Evaluator: ________________________________

| Item | Completed |
| --- | --- |
| Elicits at least 4 of the following HPI (PC2):   - Onset of the event – 3 hours - Provocation – food - Palliation – nothing - Quality of the pain - sharp - Region – epigastric - Radiation – RUQ - Severity – 8/10 - Time - 2 weeks intermittently |  |
| Elicits at least 4 of the following history (PC2, PC5, SBP3):   - PMHx – GERD - PSHx – Appy - FHx – Mom w/ HTN - Meds – Prevacid - Allergies – NKDA |  |
| Elicits at least 5 of the following ROS (PC2):   - Low grade fever - Chills - Nausea - Emesis x 1 - No weight loss - No bowel symptoms (No diarrhea, no constipation, no melana, no hematochezia - No urinary symptoms (no dysuria, no frequency, no hematuria) - No MSK symptoms (no myalgias, no joint pain, no back pain) - No neurologic symptoms (no headaches, no weakness/numbness, no LOC, no dizziness, no seizure) - No psychiatric symptoms (no SI/HI, no substance abuse, no depression, no anxiety) - All other ROS negative |  |
| Elicits cardiac/pulmonary and abdominal exam (PC2):   - For abdominal exam – does 2 of the following:   - Inspection   - Auscultation   - Percussion   - Palpation |  |
| Demonstrates behavior that conveys caring, honesty, genuine interest and tolerance when interacting with the patient (PROF1): |  |
| Establishes rapport with and demonstrate empathy toward the patient; Listens effectively to the patient (ICS1): |  |
| TOTAL SCORE | /6 |

**Station #1B Checklist – Patient Presentation**

Resident: _________________________________ Evaluator: ________________________________

| Item | Completed |
| --- | --- |
| Presents **comprehensive** history of present illness in a concise and **organized** fashion (PC2) |  |
| Presents **comprehensive** past medical/surgical hx/family hx/meds/allergies/ROS in a concise and organized fashion (PC2) |  |
| Constructs a list of potential diagnoses based on chief complaint/initial assessment (PC4) |  |
| Determines the necessity of diagnostic studies (PC3) |  |
| Offers a plan that is coherent regarding the situation (ICS2) |  |
| TOTAL SCORE | /5 |

**Station #2 Checklist – Patient Simulation**

Resident: _________________________________ Evaluator: ________________________________

| Item | Completed |
| --- | --- |
| Recognizes abnormal vital signs during initial encounter (PC1) |  |
| Elicits HPI (PC2) |  |
| Elicits past medical history (PC2) |  |
| Elicits past medications (SBP3) |  |
| Elicits allergies to medications (PC5) |  |
| Completes a focused physical exam (PC2) |  |
| Reevaluates the patient following adenosine/intervention (PC6) |  |
| Sends patient home or to observation for monitoring (PC7) |  |
| Able to multitask a single patient amidst distractions (PC8) |  |
| Demonstrates a caring nature to patient (PROF 1) |  |
| Establishes rapport with the patient (ICS 1) |  |
| Listens effectively to patients (ICS 1) |  |
| TOTAL SCORE | /12 |

Chronology of Events:

**Station #3 Checklist – Venous Vascular Access**

Resident: _________________________________ Evaluator: ________________________________

| Item | Completed |
| --- | --- |
| Uses Universal Precautions (PC9)   - Puts gloves on - No needle recapping |  |
| Demonstrates clean technique (PC9)   - Cleans/preps area |  |
| Successfully performs a venipuncture (PC 14) |  |
| Successfully places a peripheral IV (PC 14) |  |
| TOTAL SCORE | /4 |

**Station #4 Checklist – Wound Management**

Resident: _________________________________ Evaluator: ________________________________

| Item | Completed |
| --- | --- |
| Uses Universal Precautions (PC9)   - Puts gloves on - No needle recapping |  |
| Performs local anesthesia using appropriate dose of local anesthetic (PC11)   - Choses and draws up 1% lidocaine with or without epi |  |
| Uses appropriate technique to provide skin to subdermal anesthesia (PC11)   - Uses chloraprep or betadine around wound edges only prior to anesthetic injection - Goes through wound edge for injection with small bore needle - Pulls back to assure not in a vessel - Slow infiltration along both sides of wound |  |
| Prepares a simple wound for suturing (PC13)   - Identifies appropriate suture material – uses non-absorbable suture - Irrigates wound |  |
| Demonstrates sterile technique (PC13)   - Drapes wound - Uses sterile gloves |  |
| Places at least 3 simple interrupted sutures successfully (PC13)   - Instrument ties - Successfully uses instruments - Square knots |  |
| TOTAL SCORE | /6 |

**Station #5 Checklist – Arterial Puncture**

Resident: _________________________________ Evaluator: ________________________________

| Item | Completed |
| --- | --- |
| Uses Universal Precautions (PC9)   - Puts gloves on - No needle recapping |  |
| Demonstrates sterile technique (PC9)   - Cleans/preps area - Drapes wound - Uses sterile gloves |  |
| Successfully performs an arterial puncture (PC 14)   - Feels for the pulse during placement, readjusts if needed - Demonstrates familiarity with supplies/kit - Does not need to include placement of the arterial line, but please encourage PGY1 to do so if time permits |  |
| TOTAL SCORE | /3 |

**Station #6 Checklist – Airway**

Resident: _________________________________ Evaluator: ________________________________

| Item | Completed |
| --- | --- |
| Uses Universal Precautions (PC9)   - Puts gloves on - Places face shield with mask on |  |
| Describes upper airway anatomy (PC10)   - Successfully answers 8 of the 9 structures correct on the diagram provided - No prompting/hints please |  |
| Performs jaw thrust successfully (PC10) |  |
| Performs chin lift successfully (PC10) |  |
| Places oral airway successfully (PC10) |  |
| Places nasopharyngeal airway successfully (PC10) |  |
| Performs BVM successfully (PC10) |  |
| Assembles laryngoscopy handle and blade together correctly |  |
| Uses left hand to attempt intubation   - PGY1 may proceed with intubation if time allows |  |
| TOTAL SCORE | /9 |
