# Supplementary material for: Early Emergency Medicine Milestone Assessment for Predicting First-Year Resident Performance
Source: MedEdPORTAL. 2024 Mar 12;20:11386. doi: 10.15766/mep_2374-8265.11386 (PMC10928014; doi:10.15766/mep_2374-8265.11386)
Supplement: Supplementary file 1 — MED Stations and Schedule.docxSample EM PGY 1 Orientation Didactic Syllabus.docxMED Checklists.docxMED Station 1 Materials.docxMED Station 2 Materials.docxMED Station 3 Materials.docxMED Station 4 Materials.docxMED Station 5 Materials.docxMED Station 6 Materials.docxMED Station 7 Materials.docxMED Performance Summary.docx [file mep_2374-8265.11386-s001.zip › K. MED Performance Summary.docx]

| **Milestone Evaluation Day Performance** | |
| --- | --- |
| Resident: | |
|  | **Score** |
| **Station 1** |  |
| - A: History/Physical Exam (PC2, PC5, PROF1, ICS1, SBP3)   - Appropriately acquired all pertinent HPI, past history and ROS (2 points)   - Performed a focused physical exam (2 points)   - Acted in a caring manner (1 point)   - Established rapport and listed effectively (1 point)   - Comments: | /6 |
| - B: Patient Presentation (PC2, PC3, PC4)   - Presented in a concise and organized fashion, including a comprehensive past medical history, meds, allergies (2 points)   - Constructed a list of potential diagnosed based on the chief complaint (1 point)   - Determined the necessity of appropriate testing (1 point)   - Appropriately offered a plan regarding the patient (1 point)   - Comments: | /5 |
|  |  |
| **Station 2** |  |
| - Patient Simulation (PC1, PC2, PC5, PC6, PC7, PC8, ICS1, SBP3, PROF1)   - Appropriately recognized the patient’s abnormal vital signs (2 points)   - Appropriately obtained the HPI, past medical history, medications and allergies (2 points)   - Completed a focused physical exam (2 points)   - Reevaluated the patient following intervention (1 point)   - Made appropriate disposition for this patient (1 point)   - Able to multitask a single patient amidst distraction (1 point)   - Demonstrated a caring nature (1 point)   - Established rapport (1 point)   - Listened effectively (1 point)   - Comments: | /12 |
|  |  |
| **Station 3** |  |
| - A: Peripheral IV Placement/Venipuncture (PC9, PC14)   - Used universal precautions (1 point)   - Demonstrated clean technique (1 point)   - Successful venipuncture (1 point)   - Successful placement of a peripheral IV (1 point)   - Comments: | /4 |
| - B: Wound Care (PC9, PC11, PC13)   - Used universal precautions which included not recapping needles (1 point)   - Performed local anesthesia using appropriate technique and dose (1 point)   - Prepared a simple wound for suturing (1 point)   - Demonstrated sterile technique (1 point)   - Placed 3 simple interrupted sutures with good technique (2 points)   - Comments: | /6 |
|  |  |
| **Station 4** |  |
| - A: Arterial Line (PC9, PC14)   - Used universal precautions (1 point)   - Demonstrated sterile technique (1 point)   - Successfully performed an arterial puncture (1 point)   - Comments: | /3 |
| - B: Airway (PC9, PC10)   - Used universal precautions (1 point)   - Able to identify upper airway anatomy (4 points)   - Performed jaw thrust, chin lift, oral airway, nasopharyngeal airway and BVM successfully (2 points)   - Assembled laryngoscopy handle/blade together correctly (1 point)   - Used proper hand placement to attempt intubation (1 point)   - Comments: | /9 |
|  |  |
| **Station 5** |  |
| - Written Exam (PC5, PC12, SBP1, SBP2, PBLI)   - 45 total questions | /45 |
|  |  |
| **MISC** |  |
| - Accountability (PROF2) | /1 |
