# Supplementary material for: Early Emergency Medicine Milestone Assessment for Predicting First-Year Resident Performance
Source: MedEdPORTAL. 2024 Mar 12;20:11386. doi: 10.15766/mep_2374-8265.11386 (PMC10928014; doi:10.15766/mep_2374-8265.11386)
Supplement: Supplementary file 1 — MED Stations and Schedule.docxSample EM PGY 1 Orientation Didactic Syllabus.docxMED Checklists.docxMED Station 1 Materials.docxMED Station 2 Materials.docxMED Station 3 Materials.docxMED Station 4 Materials.docxMED Station 5 Materials.docxMED Station 6 Materials.docxMED Station 7 Materials.docxMED Performance Summary.docx [file mep_2374-8265.11386-s001.zip › D. MED Station 1 Materials.docx]

| **Appendix D: Station #1A History and Physical Exam Simulation Case**  **SIMULATION CASE TITLE: Station #1A – History and Physical Exam**  **AUTHORS: Danielle Turner-Lawrence, MD**  **LEARNER AUDIENCE: PGY1 residents** | |
| --- | --- |
| **PATIENT NAME: Khalil More**  **PATIENT AGE: 29**  **CHIEF COMPLAINT: “My belly hurts”**  **PHYSICAL SETTING: Emergency Department** | |
|  | |
| **Brief Narrative Description of Case** | *29yo patient presenting to the emergency department with chief complaint of abdominal pain. The overall learner goal for the trainee is to obtain a focused history and physical within the allotted time frame. Trainees are instructed to tell the patient they will go discuss the case with their attending once they have finished.* |
| **Primary Learning Objectives** | *1. Perform a focused history and physical exam*  *2. Ask patient for drug allergies*  *3. Demonstrate behavior that conveys caring, honesty, genuine interest and tolerance when interacting with a diverse population of patients and families*  *4. Establish a rapport with and demonstrate empathy towards patients and their families; listens effectively to patients and their families*  *5. Review medications with patients* |
| **Critical Actions** | *1. Elicits a focused history of present illness*  *2. Reviews past medical, surgical, and family history, medications, and allergies with patient*  *3. Elicits at least 5 review of systems from patient*  *4. Elicits a cardiac, pulmonary and abdominal exam*  *5. Demonstrates behavior that conveys caring, honesty, genuine interest and tolerance when interacting with the patient*  *6. Establishes rapport with and demonstrates empathy toward the patient; listens effective to the patient* |
| **Learner Preparation or Prework** | *Learners are given the primary objectives and a clinical vignette immediately prior to the case.* |

| **INITIAL PRESENTATION** | | | |
| --- | --- | --- | --- |
| **Initial Vital Signs** | **Temp:** 37.2C  **HR:** 80  **BP:** 120/70  **RR:** 14  **O2 sat:** 100% | | |
| **Overall Setting and Appearance** | *Upon entering the room, learners will see the standardized patient lying in a stretcher, appearing uncomfortable and holding their abdomen.* | | |
| **Standardized Participants (and Their Roles in the Room at Case Start)** | *The standardized patient is present at the beginning of the simulation, and they act as the patient for the remainder of the case. They are typically played by a chief resident. They will be complaining of generalized abdominal pain and will answer specific questions when elicited by the learner.* | | |
| **HPI** | *The following information must be asked, none volunteered by the patient except a general statement of abdominal pain.*  *Onset – 3 hours ago*  *Provoking – food makes it worse*  *Palliative – nothing makes it better*  *Quality – sharp*  *Location – epigastric region*  *Radiation – RUQ*  *Severity – 8/10*  *Timing – 2 weeks, intermittently*  *ROS positives: low grade fever, chills, nausea/emesis x1*  *ROS negatives: no weight loss, diarrhea, constipation, melena, hematochezia, urinary symptoms, MSK symptoms, neurological symptoms or psychiatric symptoms; all other ROS is negative* | | |
| **Past Medical/Surgical History** | **Medications** | **Allergies** | **Family History** |
| GERD  Appendectomy | Prevacid 15 mg QD PO | NKDA | Mom with HTN |
| **Physical Examination** | | | |
| **General** | Appears stated age, no acute distress, appears uncomfortable | | |
| **HEENT** | Negative | | |
| **Neck** | Negative | | |
| **Lungs** | CTA BL | | |
| **Cardiovascular** | RRR | | |
| **Abdomen** | Soft, ND, epigastric and RUQ moderately tender to palpation, voluntary guarding, no rebound, no rigidity, normal BS | | |
| **Neurological** | Normal motor, strength and cranial nerves | | |
| **Skin** | Dry, warm | | |
| **GU** | Deferred – may tell learner it is normal and hemoccult is negative if asked | | |
| **Psychiatric** | Negative | | |

**Ideal Scenario Flow**

*The learner will enter the room to find the simulated patient lying in a stretcher, appearing mildly uncomfortable and holding their abdomen. They will introduce themselves to the patient and treat the patient with empathy and respect. They will elicit a focused history and the patient will only provide information for what is asked. They will then perform a focused physical exam including cardiovascular, pulmonary and abdomen. They will then inform the patient that they will discuss the case with the attending and dismiss themselves from the room. Throughout the encounter, the learner will listen effectively.*

**Anticipated Management Mistakes**

This simulated case does not require any management, so no management mistakes are anticipated.

**Station #1A – History and Physical Exam**

PGY1 Instructions:

Please perform a complete history and physical exam relevant for the patient’s complaint. Once complete, you do not need to give the patient any explanation. Politely excuse yourself to “discuss the case with the attending”.

Level 1 Milestone Objectives:

Performance of Focused History and PE – Patient Care #2: Performs and communicates a reliable, comprehensive history and physical exam

Pharmacotherapy – Patient Care #5: Consistently ask patients for drug allergies

Professionalism #1 – Professional Values: Demonstrates behavior that conveys caring, honesty, genuine interest and tolerance when interacting with a diverse population of patients and families

Interpersonal Communication Skills - Patient Centered Communication #1: Establishes rapport with and demonstrates empathy towards patients and their families; Listens effectively to patients and their families

Systems Based Practice #3 – Technology: Reviews medications for patients

**Station #1B - Presentation**

PGY1 Instructions:

Please present the Station #1A patient to your attending. Be sure to include all parts of the history, physical exam, your assessment, differential diagnosis and plan for the patient in an organized fashion.

Level 1 Milestone Objectives:

Performance of Focused History and PE – Patient Care #2: Performs and communicates a reliable, comprehensive history and physical exam

Diagnostic Studies – Patient Care #3: Determines the necessity of diagnostic tests

Diagnosis – Patient Care #4: Constructs a list of potential diagnoses based on chief complaint and initial assessment

**Station #1A &1B –Evaluator Instructions**

Station #1A Instructions: You will be the patient stationed in classroom 1 and should memorize the patient’s information. Please allow the trainee to perform a history and do as much as a physical exam as you are comfortable with. Please be consistent in your responses. They have 5 minutes to complete this task. Please fill out the checklist after the trainee has left and turn in all checklists at the end of the day.

Station #1B Instructions: You will act as an attending and be stationed in classroom 2. The trainee will present the patient to you, including differential diagnoses and plan. Please fill out the checklist after the trainee has left and turn in all checklists at the end of the day. Do not give any real-time feedback.
