# Supplementary material for: Early Emergency Medicine Milestone Assessment for Predicting First-Year Resident Performance
Source: MedEdPORTAL. 2024 Mar 12;20:11386. doi: 10.15766/mep_2374-8265.11386 (PMC10928014; doi:10.15766/mep_2374-8265.11386)

| **Appendix E: Station #2 Patient Simulation Case**  **SIMULATION CASE TITLE: Station #2 – Patient Simulation**  **AUTHORS: Danielle Turner-Lawrence, MD**  **LEARNER AUDIENCE: PGY1 Residents** | |
| --- | --- |
| **PATIENT NAME: Kristin Dale**  **PATIENT AGE: 25**  **CHIEF COMPLAINT: Heart racing**  **PHYSICAL SETTING: Emergency Department** | |
|  | |
| **Brief Narrative Description of Case** | *25yo F patient is presenting to the emergency department with a chief complaint of her heart racing. Overall learner goals for this patient are to obtain a focused history and physical and appropriately medically manage the patient.* |
| **Primary Learning Objectives** | *1. Recognize abnormal vital signs*  *2. Perform and communicate a reliable, comprehensible history and physical exam*  *3. Asks patients for drug allergies*  *4. Recognizes need for patient re-evaluation*  *5. Describes basic resources available for care of emergency department patient*  *6. Demonstrates behavior that conveys caring, honesty, genuine interest and tolerance when interacting with a diverse population of patients and families*  *7. Establishes apport with and demonstrates empathy towards patients and their families; listens effective to patients and their families*  *8. Reviews medications with patients* |
| **Critical Actions** | *1. Recognizes abnormal vital signs during initial encounter*  *2. Elicits HPI*  *3. Elicits past medical history*  *4. Elicits past medications*  *5. Elicits allergies to medications*  *6. Completes a focused physical exam*  *7. Re-evaluates the patient following adenosine/intervention*  *8. Sends patient home or to observation for monitoring*  *9. Able to multitask a single patient amidst distractions*  *10. Demonstrates a caring nature to patient*  *11. Establishes rapport with patient*  *12. Listens effective to the patient* |
| **Learner Preparation or Prework** | *Learners are given a clinical vignette with learning objectives immediately prior to the start of the case. They are instructed to obtain a focused history and physical, medically manage the patient and identify a disposition.* |

| **INITIAL PRESENTATION** | | | |
| --- | --- | --- | --- |
| **Initial Vital Signs** | **HR:** 180  **BP:** 115/62  **RR:** 18  **O2 sat:** 99% | | |
| **Overall Setting and Appearance** | *Upon entering the room, learners will encounter a Laudrel SimMan laying on a stretcher. Faculty in another room will be voicing the patient. There will be a “nurse” standing at the bedside waiting for instructions from the trainee. The trainee will be handed a clinical vignette that has patient information, chief complaint and initial vital signs.* | | |
| **Standardized Participants (and Their Roles in the Room at Case Start)** | *The SimMan is voiced by faculty in another room, acting as the simulated patient.*  *There will be a faculty member in the room acting as a nurse, waiting for instructions from the learner.*  *There will be another faculty member in a different room observing and scoring the learner based on the checklist.* | | |
| **HPI** | *The following is presented by the patient without prompting: 24yo F presents to the emergency department with chief complaint of heart racing. She was at home when she had just finished dinner of chicken, corn, mashed potatoes, and a coke. She suddenly developed a vague chest pressure and felt like her heart was racing. She is not short of breath. This has happened once prior, but she never knew what it was from.*  *Further ROS asked by the learner will all be unremarkable. Negative social history including illicit drugs.* | | |
| **Past Medical/Surgical History** | **Medications** | **Allergies** | **Family History** |
| None | None | NKDA | None |
| **Physical Examination** | | | |
| **General** | Patient is talking in NAD | | |
| **HEENT** | NCAT, face without tenderness or lesions, PERRLA, TM clear bilaterally, nose unremarkable, oropharynx clear | | |
| **Neck** | No JVD or tenderness | | |
| **Lungs** | CTA BL | | |
| **Cardiovascular** | Regular, tachycardic, no murmurs, rubs or gallops | | |
| **Abdomen** | Soft, NDNT | | |
| **Neurological** | GCS 15 | | |
| **Skin** | Dry, warm | | |
| **GU** | Normal | | |
| **Psychiatric** | Normal | | |

| **INSTRUCTOR NOTES - CHANGES AND CASE BRANCH POINTS**  *This section should be a list with detailed description of each step that may happen during the case. If medications are given, what is the response? Do changes occur at certain time points? Should the nurse or other participant prompt the learners at given points? Should new actors or participants enter, and when? Are there specific things the patient will say or do at given times? There are a few examples given, but it is expected that most cases will have many more changes and potential branch points.*  *If you have a more complex branching algorithm than can be accommodated by the structure below, feel free to replace this section with your own. Review some recent simulation publications on MedEdPORTAL for examples.* | | |
| --- | --- | --- |
| **Intervention / Time Point** | **Change in Case** | **Additional Information** |
| *Placed on monitor, IV, O2* | *If learner does not ask this of nurse within first 5 minutes, nurse will prompt learner to put patient on monitor* | *RN states “Doctor, would you like this patient on the monitor?* |
| *Orders ECG* | Nurse gives learner ECG, should recognize SVT |  |
| *Performs vagal maneuvers* | *No change in rhythm* |  |
| *Gives 6 mg of adenosine* | *Conversion of SVT to NSR* |  |
| Orders post-adenosine ECG | Nurse gives learner ECG, should recognize NSR |  |
| Orders labs/iaing | Nurse will provide learner with specific lab values and image of CXR when asked | See attached lab values and images in Appendix E |
| Disposition | Learner can either dispo home or admit to observation | Prompted by patient if the learner does not state intervention, patient states “Can I go home now doctor?” |

**Ideal Scenario Flow**

*The learner enters the room to see the SimMan laying on a stretcher. They immediately ask the nurse to place the patient on the bedside monitors and get an IV. They immediately recognize the patient is tachycardic. They ask the nurse for an ECG. While the nurse is getting an ECG, the learner elicits a focused history and physical exam. Once seeing the ECG, they recognize that the patient is in SVT. They attempt a vagal maneuver, but this does not work. They instruct the nurse to give 6 mg of adenosine. Prior to administration of the medication, they explain to the patient what is happening and what they might feel when getting the medication. After administration of adenosine, they recognize that the patient’s heart rate has normalized. They re-evaluate the patient and ask the nurse for a repeat ECG, recognizing that it is now normal sinus rhythm. They order labs and chest X-ray, recognizing no abnormalities. They inform the patient of their plan for disposition which can be discharge home with primary care follow up or admission to observation.*

**Anticipated Management Mistakes**

*For example:*

1. *Difficulty with bedside monitors: Sometimes the bedside monitors are not working in real time and will not show vitals. This could be modified by having the nurse verbalize current and changes in vitals to the learner.*
2. *Unable to recognize dosage of adenosine: Some of our learners did not know the exact dose of adenosine to be given. This was modified by the nurse suggesting the dose or stating that the pharmacy called and recommended a dose of 6mg.*
3. *Failure to recognize the need for monitors: Some of our learners did not immediately place the patient on monitors to see the abnormal vital signs. After 5 minutes have passed, the nurse would prompt the learner to place the patient on the monitors.*
4. *Uncertainty about indicating disposition: Many of our learners would forget to give the patient a disposition at the end of the case. This was modified by the patient prompting the learner by asking to be sent home.*

**Station #2 – Patient Simulation**

PGY1 Instructions:

Please perform a complete history and physical exam on the simulated patient just as you would in the Emergency Department (ED). Carry out diagnosis, treatment and disposition. Your nurse is available for assistance and will carry out your orders and obtain any needed supplies. Please address the simulated patient and their family as you would any other patient in the ED.

You will be notified overhead once the case is completed.

Level 1 Milestone Objectives:

Emergency Stabilization – Patient Care #1: Recognizes abnormal vital signs

Performance of Focused History and PE – Patient Care #2: Performs and communicates a reliable, comprehensive history and physical exam

Pharmacotherapy – Patient Care #5: Consistently asks patients for drug allergies

Observation and Reassessment – Patient Care #6: Recognizes the need for patient re-evaluation

Disposition – Patient Care #7: Describes basic resources available for care of the emergency department patient

Multi-tasking – Patient Care #8: Manages a single patient amidst distractions

Professionalism #1 – Professional Values: Demonstrates behavior that conveys caring, honesty, genuine interest and tolerance when interacting with a diverse population of patients and families

Interpersonal Communication Skills - Patient Centered Communication #1: Establishes rapport with and demonstrates empathy towards patients and their families; Listens effectively to patients and their families

Technology – Systems Based Practice #3: Reviews medications for patients

**Station #2 – Patient Simulation Evaluator Instructions**

Evaluator Instructions:

You will be stationed in the simulation lab. Trainees have 10 minutes to complete the patient scenario after they have reviewed the patient clinical vignette on the door.

Faculty running Laerdal SimMan: You will be at the computer and running the SVT case. Please start the timer when the trainee has entered the room. Answer the trainee questions as the SimMan based on the patient information. Please be consistent with your responses. The initial patient rhythm will be SVT. Once the adenosine is pushed, they should convert to a normal sinus rhythm. See branch scenario points for changes in patient status.

Nurse role-player: You will be next to the patient simulator when the trainee enters the room. Please “hook” the patient up to the monitor and follow instructions set out by the trainee. If prompted to do vagal maneuvers, ask the trainee to clarify what this means. If prompted to give medications, ask about dosages and route. You will have a set of labs/CXR/ECG, please provide these to the trainee when asked.

Evaluator: Please observe the trainee in the patient simulation scenario, taking notes of key events. Please fill out the checklist and turn in all checklists at the end of the day. Do not give any real time feedback or prompt the trainee if they are stuck.

**Station #2 – Patient Simulation Workup Results**

Lab Results:

- CBC
  - WBC: 10.2 /mm^3^
  - Hgb 13.1 g/dL
  - Plts 450 /mm^3^
- Chem-7
  - Na+ 141 mEq/L
  - K+ 4.1 mEq/L
  - HCO3- 20 mEq/L
  - Cl- 103 mEq/L
  - Glucose 72 mg/dL
  - BUN 11 mg/dL
  - Create 0.9 mg/dL
- Urine Drug Screen
  - Amphetamines: negative
  - Cocaine: negative
  - Opiates: negative
  - Benzodiazepines: negative
- ETOH
  - <10 mg/dL
- BNP: 690
- Cardiac enzymes: normal
- TSH, free T4, T3: WNL

Pre-adenosine ECG:


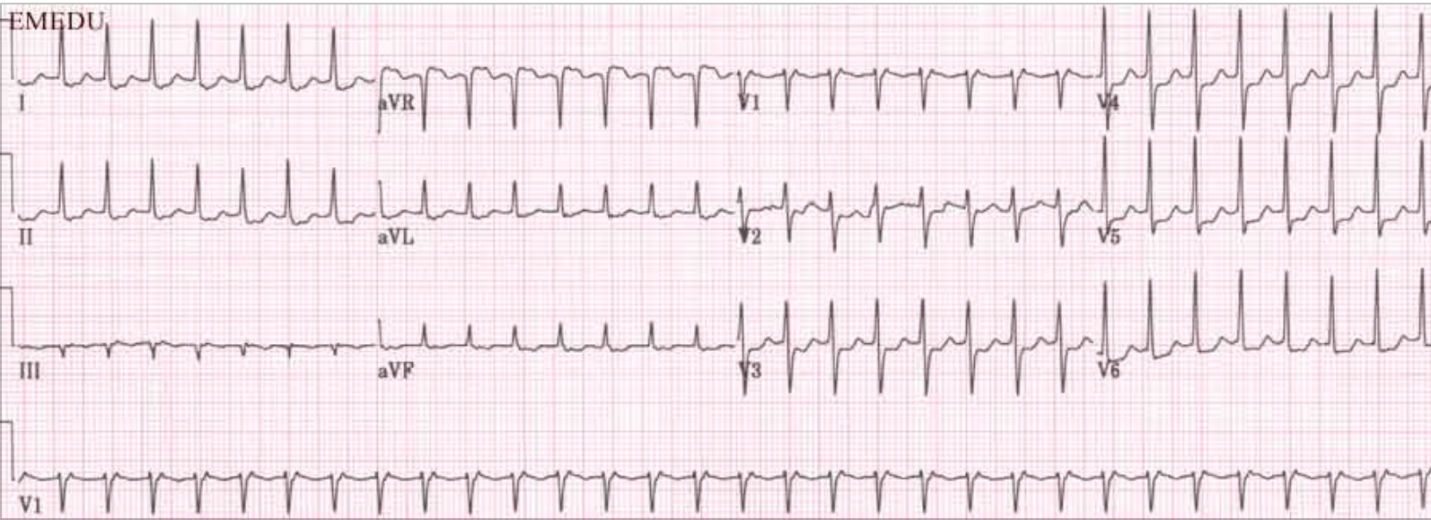


Post-adenosine ECG:


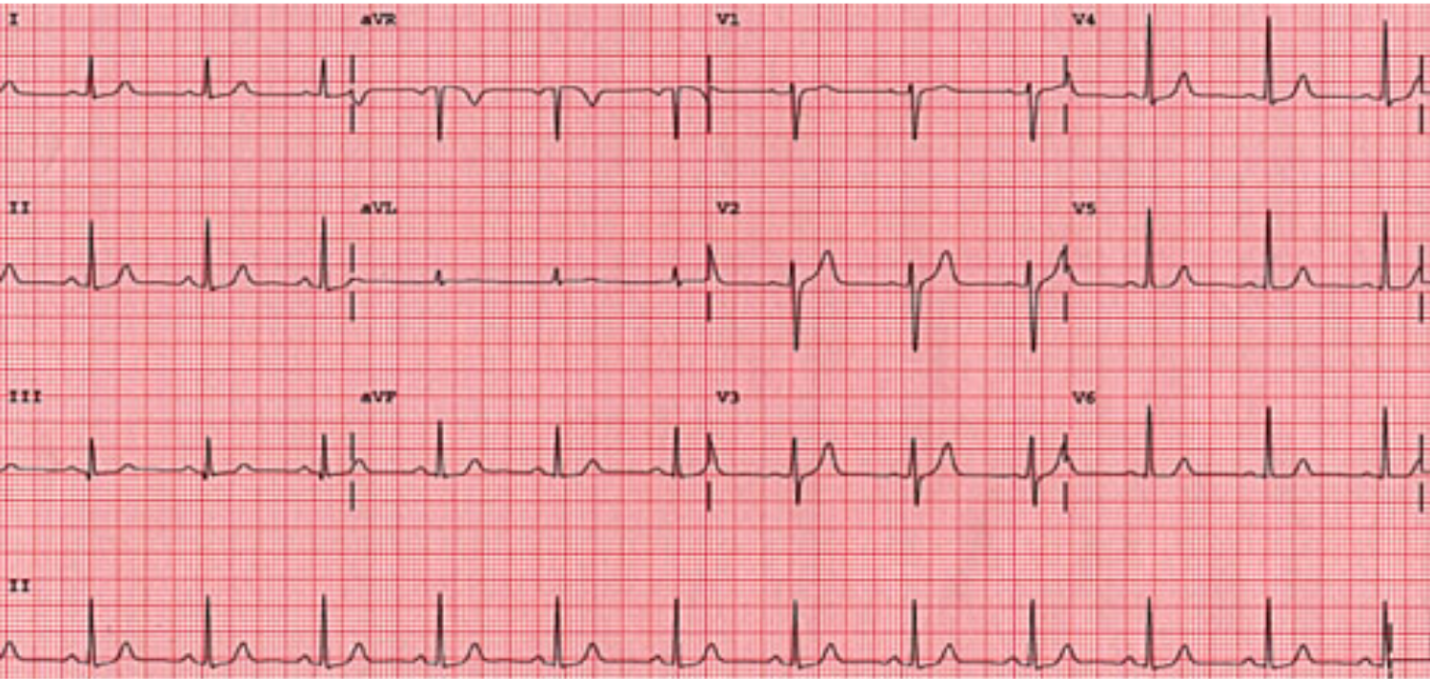


Chest X-ray:


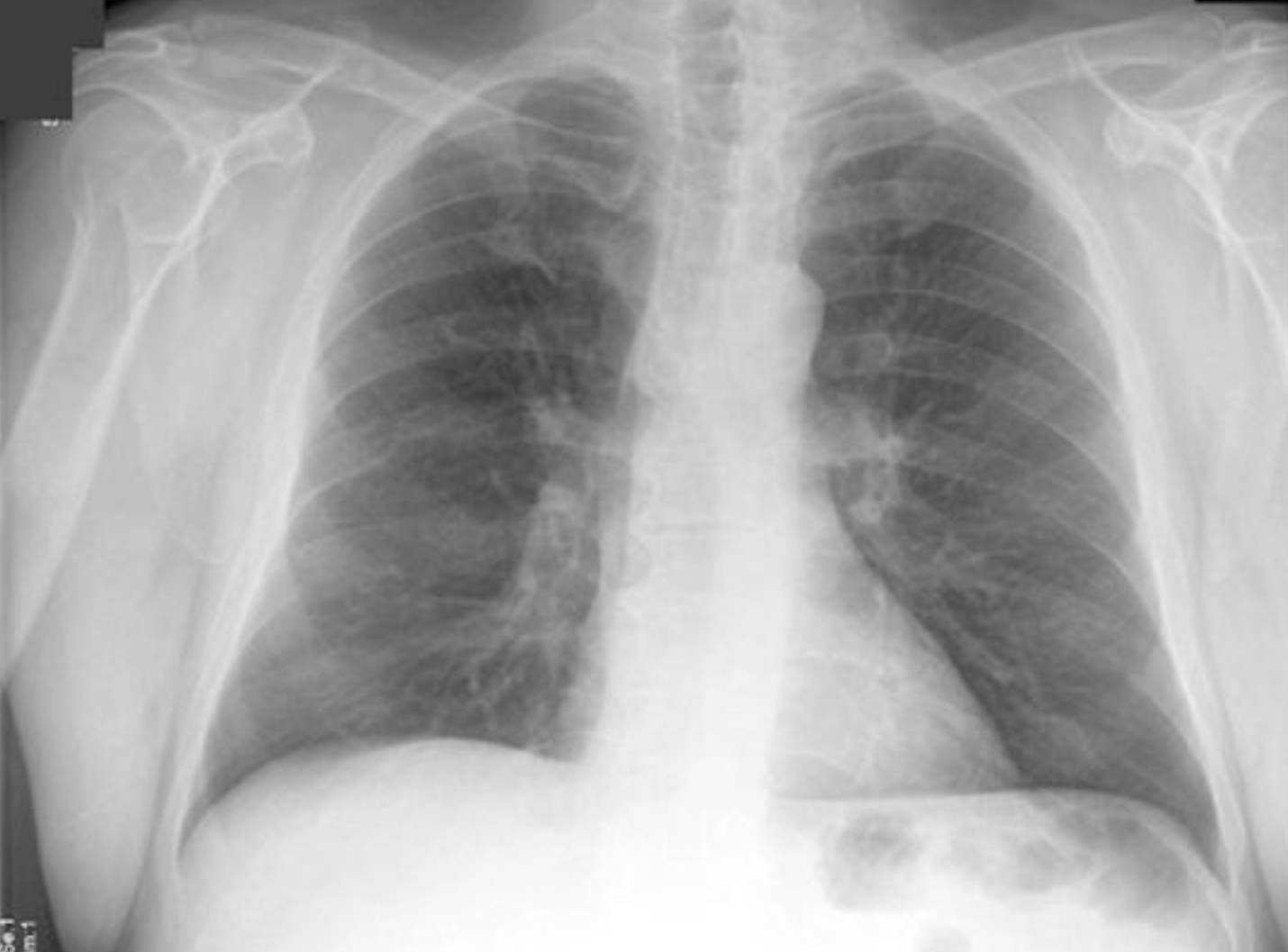

Supplement: Supplementary file 1 — MED Stations and Schedule.docxSample EM PGY 1 Orientation Didactic Syllabus.docxMED Checklists.docxMED Station 1 Materials.docxMED Station 2 Materials.docxMED Station 3 Materials.docxMED Station 4 Materials.docxMED Station 5 Materials.docxMED Station 6 Materials.docxMED Station 7 Materials.docxMED Performance Summary.docx [file mep_2374-8265.11386-s001.zip › E. MED Station 2 Materials.docx]
