# Supplementary material for: Early Emergency Medicine Milestone Assessment for Predicting First-Year Resident Performance
Source: MedEdPORTAL. 2024 Mar 12;20:11386. doi: 10.15766/mep_2374-8265.11386 (PMC10928014; doi:10.15766/mep_2374-8265.11386)
Supplement: Supplementary file 1 — MED Stations and Schedule.docxSample EM PGY 1 Orientation Didactic Syllabus.docxMED Checklists.docxMED Station 1 Materials.docxMED Station 2 Materials.docxMED Station 3 Materials.docxMED Station 4 Materials.docxMED Station 5 Materials.docxMED Station 6 Materials.docxMED Station 7 Materials.docxMED Performance Summary.docx [file mep_2374-8265.11386-s001.zip › F. MED Station 3 Materials.docx]

**Station #3 – Venous Vascular Access**

PGY1 Instructions:

Please use the trainer to place an intravenous (IV) line with the supplies provided. You should properly prep the mannequin and perform a venipuncture with placement of an IV as you would in a real patient

Level 1 Milestone Objectives:

General Approach to Procedures – Patient Care #9: Identifies pertinent anatomy and physiology for a specific procedure; Uses appropriate Universal Precautions

Vascular Access – Patient Care #14: Performs a venipuncture; Places a peripheral intravenous line

**Station #3 – Venous Vascular Access Evaluator Instructions**

Evaluator Instructions: You will be stationed in the simulation area. Trainees have 5 minutes for this station. There will be a peripheral IV trainer and IV supplies. The trainee will prep the skin and will need to successfully place a peripheral IV. Please fill out the checklist after the trainee has left. Turn in all checklists at the end of the day. Do not provide any real-time feedback.
