# Supplementary material for: Early Emergency Medicine Milestone Assessment for Predicting First-Year Resident Performance
Source: MedEdPORTAL. 2024 Mar 12;20:11386. doi: 10.15766/mep_2374-8265.11386 (PMC10928014; doi:10.15766/mep_2374-8265.11386)
Supplement: Supplementary file 1 — MED Stations and Schedule.docxSample EM PGY 1 Orientation Didactic Syllabus.docxMED Checklists.docxMED Station 1 Materials.docxMED Station 2 Materials.docxMED Station 3 Materials.docxMED Station 4 Materials.docxMED Station 5 Materials.docxMED Station 6 Materials.docxMED Station 7 Materials.docxMED Performance Summary.docx [file mep_2374-8265.11386-s001.zip › G. MED Station 4 Materials.docx]

**Station #4 – Wound Management**

PGY1 Instructions:

Please demonstrate wound management for the pig flank provided as you would on a real patient. This should include, but is not limited to the following:

- Drape wound in a sterile fashion
- Anesthetize wound
- Prepare wound for laceration repair
- Place 3 simple interrupted sutures

Level 1 Milestone Objectives:

General Approach to Procedures – Patient Care #9: Identifies pertinent anatomy and physiology for a specific procedure; Uses appropriate Universal Precautions

Anesthesia and Acute Pain Management – Patient Care #11:  Performs local anesthesia using appropriate doses of local anesthetic and appropriate technique to provide skin to sub-dermal anesthesia for procedures

Wound Management – Patient Care #13: Prepares a simple wound for suturing (identify appropriate suture material, anesthetize wound and irrigate); Demonstrates sterile technique; Places a simple interrupted suture

**Station #4 – Wound Management Evaluator Instructions**

Evaluator Instructions: You will be stationed in the simulation area. Trainees have 10 minutes for this station. There will be pork at this station, please make lacerations in the pork skin for the trainees to suture. Observe the trainees prep the wound in a sterile fashion, inject lidocaine, and place 3 simple interrupted sutures. Please fill out the checklist after the trainee has left. Turn in all checklists at the end of the day. Do not provide any real-time feedback.
