# Supplementary material for: Early Emergency Medicine Milestone Assessment for Predicting First-Year Resident Performance
Source: MedEdPORTAL. 2024 Mar 12;20:11386. doi: 10.15766/mep_2374-8265.11386 (PMC10928014; doi:10.15766/mep_2374-8265.11386)
Supplement: Supplementary file 1 — MED Stations and Schedule.docxSample EM PGY 1 Orientation Didactic Syllabus.docxMED Checklists.docxMED Station 1 Materials.docxMED Station 2 Materials.docxMED Station 3 Materials.docxMED Station 4 Materials.docxMED Station 5 Materials.docxMED Station 6 Materials.docxMED Station 7 Materials.docxMED Performance Summary.docx [file mep_2374-8265.11386-s001.zip › H. MED Station 5 Materials.docx]

**Station #5 – Arterial Puncture**

PGY1 Instructions:

Please use the trainer to perform a radial artery puncture with the supplies provided. You should properly drape the mannequin and perform the arterial puncture as you would on a real patient.

Level 1 Milestone Objectives:

General Approach to Procedures – Patient Care #9: Identifies pertinent anatomy and physiology for a specific procedure; Uses appropriate Universal Precautions.

Vascular Access – Patient Care #14: Performs an arterial puncture

**Station #5 – Arterial Puncture Evaluator Instructions**

Evaluator Instructions: You will be stationed in the simulation area. Trainees have 5 minutes for this station. There will be an arterial line trainer and radial arterial line kits. Please observe the learner prep the area utilizing sterile technique and successfully cannulate the artery. Please fill out the checklist after the trainee has left. Turn in all checklists at the end of the day. Do not provide any real-time feedback.
