# Supplementary material for: Early Emergency Medicine Milestone Assessment for Predicting First-Year Resident Performance
Source: MedEdPORTAL. 2024 Mar 12;20:11386. doi: 10.15766/mep_2374-8265.11386 (PMC10928014; doi:10.15766/mep_2374-8265.11386)
Supplement: Supplementary file 1 — MED Stations and Schedule.docxSample EM PGY 1 Orientation Didactic Syllabus.docxMED Checklists.docxMED Station 1 Materials.docxMED Station 2 Materials.docxMED Station 3 Materials.docxMED Station 4 Materials.docxMED Station 5 Materials.docxMED Station 6 Materials.docxMED Station 7 Materials.docxMED Performance Summary.docx [file mep_2374-8265.11386-s001.zip › I. MED Station 6 Materials.docx]

**Station #6 – Airway**

PGY1 Instructions:

Please perform the following airway maneuvers:

1. Correctly demonstrate a jaw thrust
2. Correctly demonstrate a chin lift
3. Placement of a nasopharyngeal (NP) airway
4. Placement of an oral airway
5. Correctly perform bag valve mask ventilation/oxygenation
6. Correctly identify the 9 structures of the upper airway on the picture provided.

Level 1 Milestone Objectives:

General Approach to Procedures – Patient Care #9: Identifies pertinent anatomy and physiology for a specific procedure; Uses appropriate Universal Precautions

Airway Management – Patient Care #10: Describes upper airway anatomy; Performs basic airway maneuvers or adjuncts (jaw thrust/chin lift/oral airway/nasopharyngeal airway) and ventilates/oxygenates patient using BVM

**Station #6 – Airway Evaluator Instructions**

Evaluator Instructions: You will be stationed in the simulation area. Trainees have 10 minutes for this station. There will be an airway mannequin, please ask the trainee to perform a jaw thrust, chin list, place a nasopharyngeal airway, place an oral airway, and perform bag mask ventilation on the mannequin. There will be a picture of airway anatomy, please ask the trainee to identify all 9 structures. Please fill out the checklist after the trainee has left. Turn in all checklists at the end of the day. Do not provide any real-time feedback.

**Anatomy Pictures (with and without answers)**

**
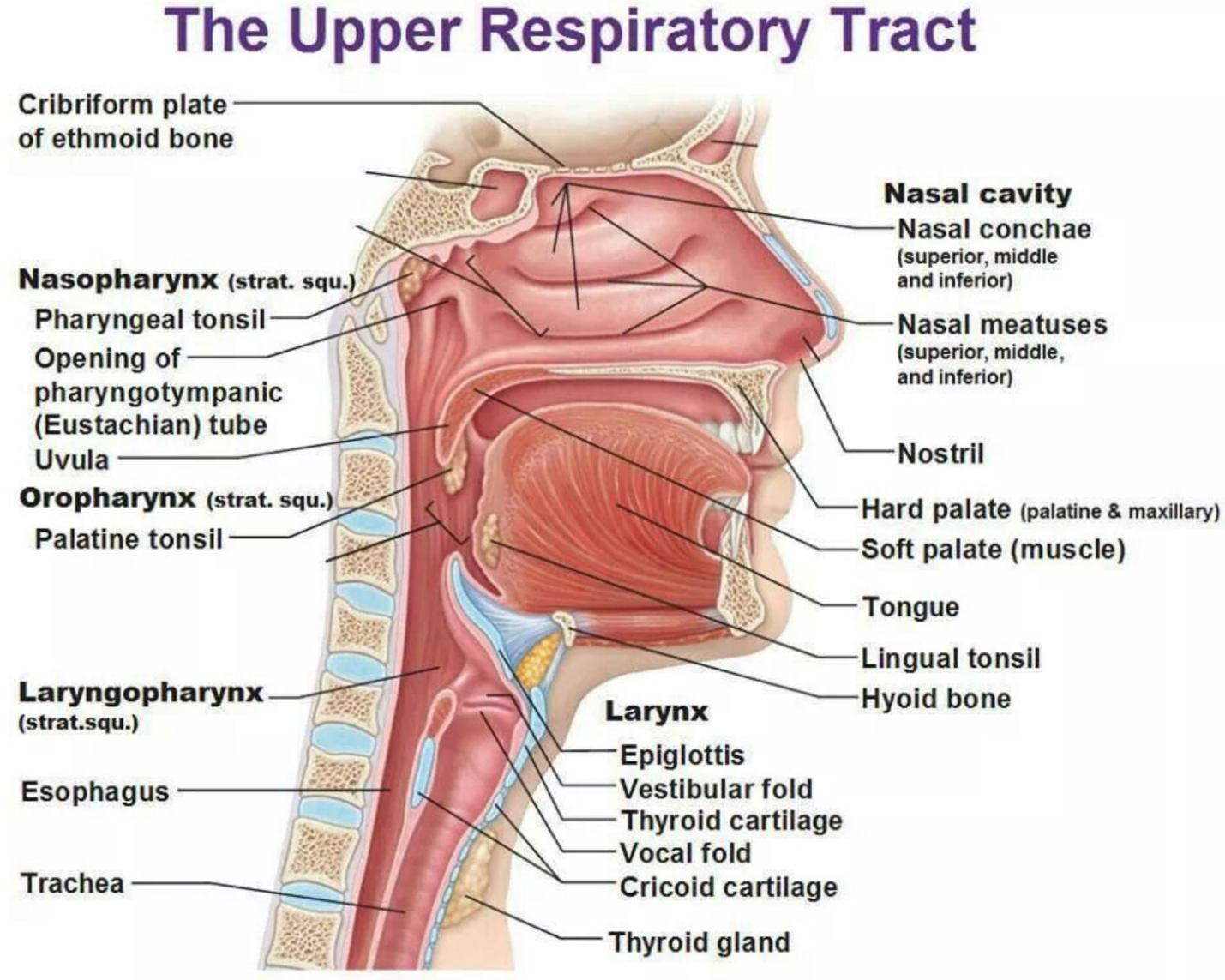
**

“Image by Uchechukwu Chukwuemeka retrieved from: <https://www.physio-pedia.com/Upper_respiratory_airways#/media/File:Upper_respiratory_system_2.jpg> on 6/27/23. Image is in the public domain.”


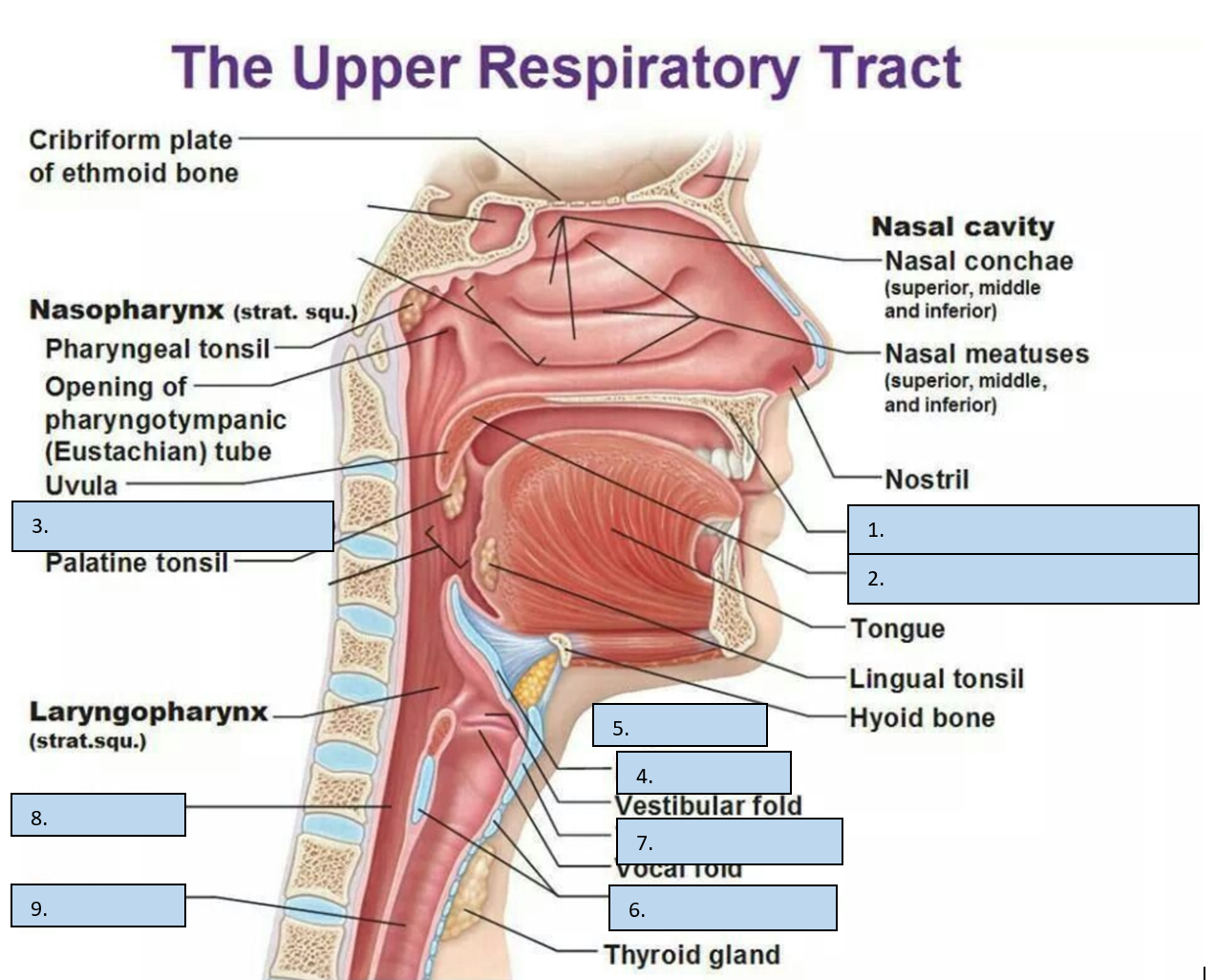


“Image by Uchechukwu Chukwuemeka retrieved from: <https://www.physio-pedia.com/Upper_respiratory_airways#/media/File:Upper_respiratory_system_2.jpg> on 6/27/23. Image is in the public domain.”

9.

6.
