# Supplementary material for: Early Emergency Medicine Milestone Assessment for Predicting First-Year Resident Performance
Source: MedEdPORTAL. 2024 Mar 12;20:11386. doi: 10.15766/mep_2374-8265.11386 (PMC10928014; doi:10.15766/mep_2374-8265.11386)
Supplement: Supplementary file 1 — MED Stations and Schedule.docxSample EM PGY 1 Orientation Didactic Syllabus.docxMED Checklists.docxMED Station 1 Materials.docxMED Station 2 Materials.docxMED Station 3 Materials.docxMED Station 4 Materials.docxMED Station 5 Materials.docxMED Station 6 Materials.docxMED Station 7 Materials.docxMED Performance Summary.docx [file mep_2374-8265.11386-s001.zip › J. MED Station 7 Materials.docx]

**Station #7 – Written Exam**

PGY1 Instructions:

Please complete the attached written exam without the help of any outside resources. Clearly mark the BEST answer on the answer key for each question. You have a total of 30 minutes to complete all questions, and if needed you may return at the end of your scheduled session to spend additional time.

Level 1 Milestone Objectives:

Pharmacotherapy – Patient Care #5: Knows the different classification of pharmacologic agents and their mechanisms of action

Wound Management – Patient Care #11: Performs local anesthesia using appropriate doses of local anesthetic

Focused Ultrasound – Patient Care #12: Describes the indications for emergency ultrasound

Medical Knowledge – MK: Demonstrated appropriate medical knowledge, for current level of training, in the care of the emergency patient.

Practice-based Performance Improvement – PBLI: Describes basic principles of evidence-based medicine

**Station #7 – Written Exam Instructions**

Evaluator Instructions: You will be stationed in a classroom. Trainees have 30 minutes to complete the written exam. You will be responsible for grading the exams once the trainees are finished, the answer key is provided. Passing is 85%. Please record scores for each trainee and turn in at the end of the day.

Resident Name: ____________________________________________ Score: _______/45

**PGY1 Milestones Evaluation Day – 2018 – Station #7**

**Instructions: Please choose the BEST single answer for each question. You are not allowed any resources and should not discuss questions or answers with classmates.**

1. When evaluating an eye complaint, when should one avoid doing tonometry with a tonopen?
a. Acute angle-closure glaucoma

b. Chronic glaucoma

c. Corneal defect

d. Hyphema

2. A 44-year-old healthy female presents to the EC with a complaint of dyspnea, chest tightness and near syncope which began approximately an hour before her arrival while working in her yard. However, while waiting to be seen her symptoms have resolved and she reports to you that she would like to leave without any workup. Her triage ECG is shown below.


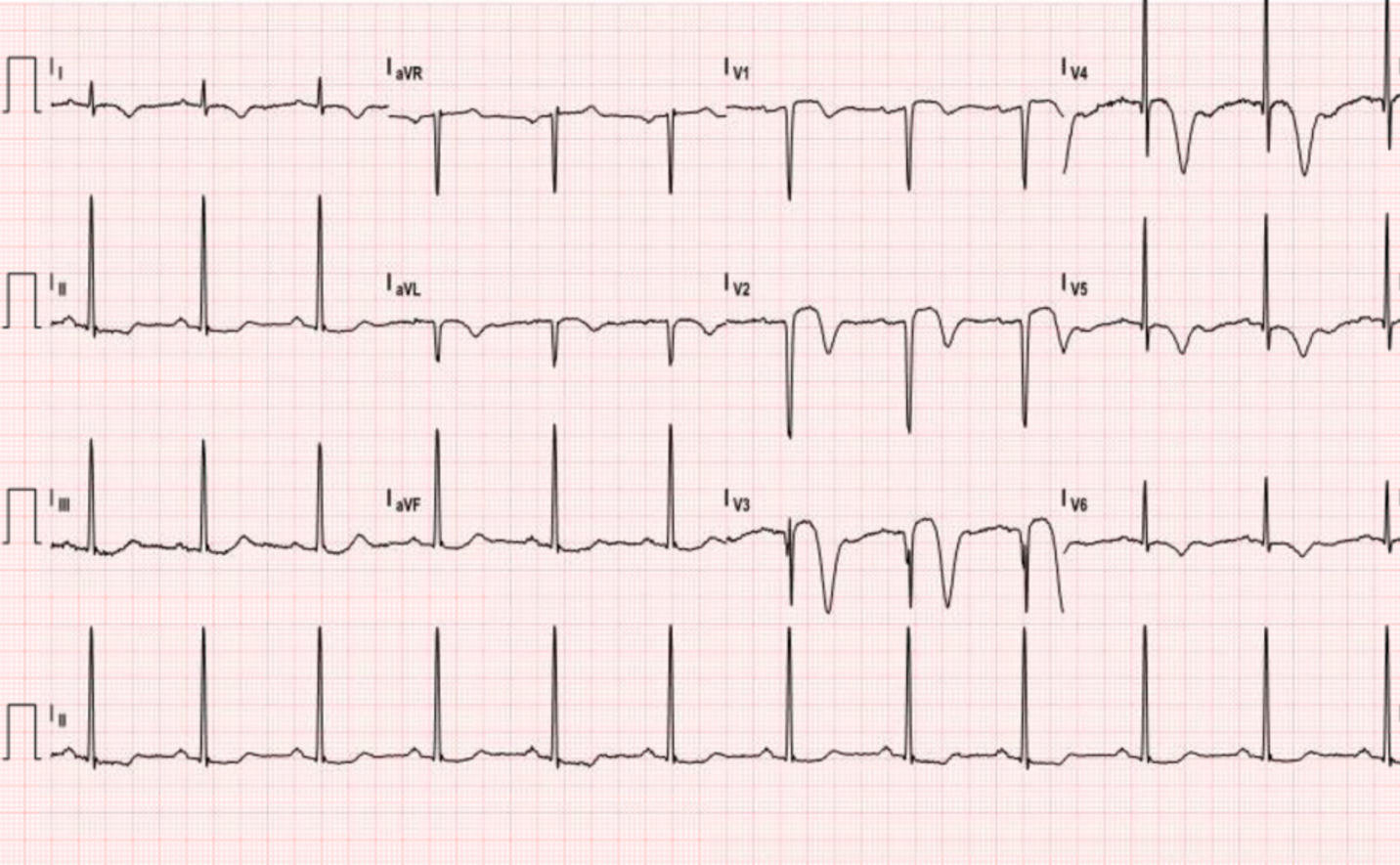


“Image by Jonathan Patane, MD and Kim Sokol, MD, retrieved from: <https://jetem.org/wellens/> on 6/27/23. Image is in the public domain.”

Your recommendation to her is that you are 95% confident that:

1. Her symptoms are due to electrolyte loss and dehydration from working in the sun and she should stay for potassium supplementation
2. Her ECG shows right axis deviation and RVH and that she needs to stay to rule out a pulmonary embolism
3. Her ECG demonstrates a concerning warning and you are believe she has a significant lesion in her LAD
4. Her ECG demonstrates a Brugada abnormality and she either needs to stay for Cardiology EP evaluation and possible AICD placement, or she should stop speed balling.
5. Her triage ECG has screened out any cardiac abnormality and she is okay to go as long as she follows up with her primary doctor within 2 days.

3. True/False: All of the following conditions require emergent ophthalmologist consultation: acute angle closure glaucoma, retinal detachment, globe rupture and central retinal artery occlusion.

4. True/False: The workup for a 6 day old infant with fever >100.5 should always include urine, blood and cerebrospinal fluid analysis.

5. What type of artifact is noted beneath the gallbladder, urinary bladder and any cyst?

a. Enhancement

b. Edge

c. Reverberation

d. Side lobe

e. Mirror

6. The following are essential components of EMS:

a. EMS providers and ambulances

b. Emergency departments

c. Cardiologist and trauma surgeons

d. Hospitals

e. All of the above.

7. How much fluid can you give in a septic pediatric patient in the first 20 minutes based on the treatment guidelines?

a. 10 ml/kg

b. 20 ml/kg

c. 40 ml/kg

d. 60 ml/kg

8. Which of the following is true regarding number needed to treat (NNT):

a. NNT is calculated based on senility

b. A large NNT means that the treatment is more effective

c. NNT is the number of patients that must be treated to obtain one additional good outcome

d. An acceptable NNT is 1000

9. Which are the standard views in the FAST exam:

a. RUQ, Subxiphoid, LUQ, Suprapubic

b. RUQ, Parasternal long axis, aorta

c. RUQ, LUQ, LLQ, RLQ

d. Visualizing Morrison’s pouch is sufficient for preliminary screening

e. Bilateral paracolic gutter views

10. The therapeutic range of lidocaine dosing for local anesthesia without epinephrine is:

a. 1-3 mg/kg

b. 3-5 mg/kg

c. 5-7 mg/kg

d. Up to 10 mg/kg

11. Etomidate (Amidate), a short acting IV agent used in RSI, has the following properties

a. Anesthetic

b. Amnestic

c. Analgesic

d. All of the above

e. None of the above

12. Which of the following work groups is most prone to violence in the Emergency Department?

a. Attending Physicians

b. Clerical staff

c. Nurses

d. Residents

e. Janitorial staff

13. The goals of monitored anesthesia care for procedural sedation include:

1. Providing pain control to enhance patient cooperation during a procedure in addition to controlling the unpleasant and painful aspects of a procedure.
2. To provide adequate control of discomfort during a procedure without cardiorespiratory compromise.
3. Monitoring of patient status during the procedure includes cardiac monitoring, pulse oximetry, blood pressure, pulse, respirations, end-tidal CO2, drugs administered including the dosage and time.
4. NPO status has not been found to be relevant for Monitored Anesthesia Care (MAC) for procedural sedation in the ED, the procedure trumps NPO status unlike if the patient was to undergo full general anesthesia.
5. All of the above.

14. A patient is noted to have nystagmus in the horizontal plane to the right. Where is the lesion causing this nystagmus located?

1. Cerebellum
2. Vestibular apparatus
3. Cortex
4. Brainstem

15. A 43-year-old woman presents with gradual increasing weakness and numbness of both legs and decreased lower extremity deep tendon reflexes. She denies back pain or fever. What is the most likely diagnosis?

1. Central cord syndrome
2. Cauda equina syndrome
3. Botulism
4. Brown-Sequard syndrome
5. Guillain-Barre syndrome

16. Which of the following rashes involves the palms and soles, is diagnosed clinically, and has a high mortality if missed?

1. Pityriasis Rosea
2. Rocky Mountain Spotted Fever
3. Erythema Nodosum
4. Erythema Infectiosum (Fifth’s Disease)
5. Roseola

17. Which of the following is true about Henoch Schonlein Purpura?

a. It is a systemic vasculitis characterized by palpable purpura

b. It is characterized by a triad of palpable purpura, joint pain, and abdominal pain

c. It may cause kidney involvement and in some cases nephrotic syndrome

d. 90% of cases occur in children under 10 years old

e. All of the above

18. What is the most common cause of Toxic Epidermal Necrolysis (TEN)?

1. Drugs
2. Mycoplasma infection
3. HIV infection
4. Herpes infection

19. Patient with large partial-thickness or full thickness burns can develop which type of shock in the acute phase?

1. Anaphylactic shock
2. Cardiogenic shock
3. Hypovolemic shock
4. Septic shock

20. Which of the following may make the diagnosis of abdominal pathology more difficult or mask serious problems?

1. Steroid use
2. Antibiotic use
3. Chemotherapy
4. Anti-inflammatories and antipyretics
5. All of the above

21. According to the SPRINT method of task management, which of the following is the correct order to complete the listed tasks?

1. Do a lumbar puncture on a 48-year-old with a headache and fever THEN discharge a 3-year-old with a sore throat THEN cardiovert an 87-year-old man with a fib with RVR and hypotension THEN see a patient with abdominal pain who has been waiting for 3 hours.
2. See patient with abdominal pain who has been waiting for 3 hours THEN cardiovert an 87-year-old man with a fib with RVR and hypotension THEN do a lumbar puncture on a 48-year-old with a headache and fever THEN discharge a 3-year-old with a sore throat.
3. Cardiovert an 87-year-old man with a fib with RVR and hypotension, do a lumbar puncture on a 48-year-old with headache and fever THEN see a patient with abdominal pain who has been waiting for 3 hours THEN discharge a 3-year-old with sore throat.
4. Cardiovert an 87-year-old man with a fib with RVR and hypotension THEN see a patient with abdominal pain who has been waiting for 3 hours THEN do a Lumbar puncture on a 48-year-old with headache and fever THEN discharge a 3-year-old with sore throat.

22. A patient presents in status epilepticus. What is your initial pharmacologic treatment?

1. Phenytoin 20 mg/kg IV infusion
2. Valproate sodium 20-40 mg/kg IV infusion
3. Diazepam 10 mg PO
4. Lorazepam 4 mg IV bolus
5. Levetiracetam 20 mg/kg IV infusion

23. A total of 25 patients present to your emergency department in the span of 15 minutes, all with similar complaints, those who can talk state they were at the subway station when they became very confused, developed bowel and bladder incontinence and lost their ability to easily control their salivation. After appropriate decontamination, which medication would you like to administer?

1. Activated charcoal
2. Atropine and pralidoxime
3. Physostigmine
4. Naloxone

24. What is the most common sign in pulmonary embolism?

a. Tachypnea

b. Tachycardia

c. Pain

d. Hypoxia

25. A 17-year-old male presents to your emergency department, brought by his mother who found him outside his home with one of his friends. His pertinent physical findings include 6mm pupils, flushed cheeks, tachycardia, dry axilla and he is warm to the touch. Most significantly, he is reaching for items in the air that don’t exist. What toxin did this patient most likely ingest?

1. Jimson weed
2. Percocet (oxycodone/acetaminophen)
3. Ethylene glycol
4. Methamphetamine

26. A 2-week-old female is brought to the ED with bilious emesis and poor feeding for the past few hours. Physical examination upon arrival reveals a fussy but nontoxic appearing infant, whose abdomen is mildly distended without evidence of rebound or guarding. Which of the following imaging studies is most likely to reveal the suspected diagnosis?

1. Abdominal ultrasound
2. Abdominal x-ray
3. Abdominal CT
4. Upper GI series
5. Barium enema

27. According to the article covered during the Landmark Studies discussion, the sensitivity of the current generation of CT scanners for detecting SAH approaches 100% up to how long after symptom onset?

a. 3 hours

b. 4.5 hours

c. 6 hours

d. 12 hours

e. 24 hours

28. In the Manny Rivers study regarding the treatment of sepsis published in 2001, the following was associated with significant reduction in mortality:

a. Early administration of antibiotics

b. Early administration of steroids

c. Early administration of Xigris

d. Early goal directed therapy

e. Early admission to the ICU

29. A 3-month-old female presents to the ED with a history of poor feeding and respiratory difficulty, which has been worsening over the prior 3 days. The child’s heart rate is 168 bpm, the respiratory rate is 72 per minute with grunting. The liver is 3 cm below the costal margin and you hear a systolic murmur. Which of the following is most likely to worsen the patient’s condition?

1. Elevate head and chest
2. Administer Oxygen
3. Furosemide (Lasix)
4. IV 20 mL/kg NS bolus
5. Digoxin

30. According to the PROPPR trial, what ratio of blood products (plasma: platelets: RBCs) achieved greater rates of hemostasis and fewer exsanguination related deaths at 24 hours?

a. 1:1:1

b. 1:2:1

c. 1:1:2

d. 1:2:2

e. 2:1:1

31. In the Nielsen TTM (Targeted Temperature Management) study of post-cardiac arrest patients, what is most accurate description of their findings?

1. 33 degree target temperature is superior
2. 36 degree target temperature is superior
3. 36 degree target temperature is non-inferior to 33 degree target temperature
4. 38 degree target temperature is superior
5. Therapeutic hypothermia is not recommended

32. True/False: MRI is required to diagnose cauda equina syndrome and should be performed before consultation to a spine surgeon.

33. A 68-year-old male with a history of hypertension and tobacco use presents complaining of sudden onset generalized abdominal pain radiating to his back. His blood pressure is currently 70/30 with a heart rate of 130 along with a respiratory rate of 22. Oral temp is 37 C. He is obese with generalized abdominal tenderness. What test would be most appropriate at this time?

1. CT abdomen/pelvis
2. Bedside abdominal ultrasound
3. Acute abdominal series
4. MRI lumbar spine with and without gadolinium

34. A 65-year-old diabetic female presents after rolling her ankle in the yard while taking the dog outside at 3am. On exam, she has an obvious deformity to her right ankle and is crying in pain. No other obvious injuries are identified. Which of the following requires that you reduce her ankle prior to obtaining an x-ray for fracture or dislocation?

1. The patient is crying in pain
2. The patient is diabetic
3. There is obvious deformity to the joint
4. There is no palpable dorsalis pedis pulse in the right foot
5. There will be a prolonged time to obtain an x-ray

35. Which of the following is LEAST LIKELY to be an appendicitis mimic?

1. Leaking aneurysm
2. Ovarian torsion
3. Psoas abscess
4. Torsed appendix testis
5. Cecal diverticulitis

36. Which statement is FALSE?

1. Up to 10% of people > age 50 with non-specific abdominal pain will prove to have an intra-abdominal cancer.
2. Pathology in non-abdominal body parts can present as abdominal pain.
3. Withholding analgesics is beneficial to prevent diagnostic confusion.
4. Abdominal pain accounts for approximately 10% of all ED visits.
5. Visceral pain is often poorly localized and difficult to characterize.

37. Which of the following is not a 'red flag' for a more potentially serious cause of abdominal pain?

1. Unexplained weight loss
2. Diffuse cramping pain
3. Advanced age
4. Immunosuppression
5. Tachycardia and hypotension

38. For a patient just intubated for respiratory failure, the respiratory therapist notifies you that they are having increased difficulty ventilating the patient and that they are now hypoxic and hypotensive. All of the following are appropriate initial management steps except:

a. Remove from ventilator and place on a bag-valve mask at 100% FiO2

b. Examine patient, assessing chest rise and breath sounds

c. Reconfirm ETT placement with a laryngoscope

d. Emergent CT scan to assess for pulmonary embolism

39. A patient in status asthmaticus presents for respiratory distress. Initial ABG shows a PCO2 of 30 mmHg (normal range 35-45 mmHg). After numerous breathing treatments, bipap and maximal therapies, repeat ABG now shows a PCO2 of 60mmHg and the patient appears more fatigued. The decision is made to intubate for which indication?

a. Failure to protect airway

b. Failure to oxygenate

c. Failure to ventilate

d. All of the above

40. A patient presents for shortness of breath. History reveals that onset was acute (in one instant or over the course of moments). This history supports all the following diagnoses except:

a. Pulmonary embolism

b. Pneumonia

c. Pneumothorax

d. Anaphylaxis

41. All of the following are suggestive of ruptured ectopic pregnancy EXCEPT?

a. Syncope

b. Shoulder pain

d. Shock

d. Vaginal discharge

42. Which of the following is an indication for admission for PID?

a. Tubo-ovarian abscess

b. Vaginal discharge

c. Dysuria

d. Vaginal bleeding

43. True/False: The 'D' in the primary survey stands for 'deformity' per ATLS.

44. True/False: According to Team STEPPS guidelines, the resident at the left of the bed should give orders to the care coordinator.

45. A 2-year-old previously healthy, vaccinated male presents to the Pediatric ED with mother after he had an episode of “jerky movements of his arms and legs” lasting about 3 minutes. Patient felt “warm” at home before this episode. Review of systems and family history are negative. On your evaluation, temperature is 102.4°F, heart rate is 130, respiratory rate is 22, SpO2 99%, GCS 15 lying in mother’s arm. No focal neurologic deficits. No skin rashes. Point of care glucose result is 95 mg/dl. Your next step should be to:

a. Give antipyretics and reassure mother

b. Give lorazepam and admit to the PICU

c. Obtain CT head

d. Intubate patient and consult neurology STAT

e. Obtain CBC, CMP, blood culture, urinalysis and urine culture, and CSF studies

Written Exam Answer Key

1. C
2. C
3. T
4. T
5. A
6. E
7. B
8. C
9. A
10. B
11. D
12. C
13. E
14. B
15. E
16. B
17. E
18. A
19. C
20. E
21. C
22. D
23. B
24. A
25. A
26. D
27. C
28. D
29. D
30. A
31. C
32. F
33. B
34. D
35. D
36. C
37. B
38. D
39. C
40. B
41. D
42. A
43. F
44. F
45. A
