# Supplementary material for: Early Emergency Medicine Milestone Assessment for Predicting First-Year Resident Performance
Source: MedEdPORTAL. 2024 Mar 12;20:11386. doi: 10.15766/mep_2374-8265.11386 (PMC10928014; doi:10.15766/mep_2374-8265.11386)
Supplement: Supplementary file 1 — MED Stations and Schedule.docxSample EM PGY 1 Orientation Didactic Syllabus.docxMED Checklists.docxMED Station 1 Materials.docxMED Station 2 Materials.docxMED Station 3 Materials.docxMED Station 4 Materials.docxMED Station 5 Materials.docxMED Station 6 Materials.docxMED Station 7 Materials.docxMED Performance Summary.docx [file mep_2374-8265.11386-s001.zip › B. Sample EM PGY 1 Orientation Didactic Syllabus.docx]

**Friday July 1**

9a-10a: Welcome & Introduction to Program Requirements

10a-11a: Introduction to Milestones

**Tuesday July 5**

830a-9a: Team STEPPS/Advanced Trauma Life Support

9a-930a: Scheduling/Shift Requirements

930a-10a: EM Foundations

10a-11a: Tour/Q&A

11a-1130a: Intro to EMS

1130a-12p: Intro to Research Study Design

**Wednesday July 6**

8a-10a: Administrative Requirements

10a-12p: Procedural Sedation

**Monday July 11**

8a-12p: Introduction to Simulation

12p-4p: Teleflex Central Venous Catheter Course

**Wednesday July 13**

8a-830a: Back Pain

830a-9a: Weak/Dizzy/Vertigo

9a-930a: Abdominal Pain

930a-10a: Poison Toxicology Patient

10a-1039a: Dermatology Review

**Thursday July 14**

8a-12p: Toxicology Curriculum for EM

**Monday July 18**

8a-9a: Splint/Suture Lab

9a-930a: Chest Pain

930a-10a: Seizure/Headache

10a-10:30a: ECG Review

10:30a-11a: Dyspnea/Vent Management

11a-1130a: Vaginal Bleeding/Pelvic Pain

1130a-12p: Sepsis/Shock

**Tuesday July 19**

10a-12p: Introduction to Milestone Evaluation Day

1-4p: Ultrasound Lecture

**Wednesday July 20**

8a-830a: Peds Fever/Sepsis

830a-9a: Neonatal Emergencies

9a-930a: Peds Abdominal Pain

930a-10a: Peds Resuscitation

10a-1030a: Peds Headache/Seizure/Head Injury

1030a-11a: Eye Complaints

11a-12p: Ophthalmology Lab

**Friday July 29**

9a-1p: Milestone Evaluation Day
