# Supplementary material for: Early Emergency Medicine Milestone Assessment for Predicting First-Year Resident Performance
Source: MedEdPORTAL. 2024 Mar 12;20:11386. doi: 10.15766/mep_2374-8265.11386 (PMC10928014; doi:10.15766/mep_2374-8265.11386)
Supplement: Supplementary file 1 — MED Stations and Schedule.docxSample EM PGY 1 Orientation Didactic Syllabus.docxMED Checklists.docxMED Station 1 Materials.docxMED Station 2 Materials.docxMED Station 3 Materials.docxMED Station 4 Materials.docxMED Station 5 Materials.docxMED Station 6 Materials.docxMED Station 7 Materials.docxMED Performance Summary.docx [file mep_2374-8265.11386-s001.zip › _Educational Summary Report_11386.pdf]

MedEdPORTAL® The AAMC Journal of Teaching and Learning Resources

Original Publication

Open Access

Early Emergency Medicine Milestone Assessment for Predicting First-Year Resident Performance

Rochelle L. Versalle, MD*, Brett R. Todd, MD, Nai-Wei Chen, PhD, Danielle E. Turner-Lawrence, MD

*Corresponding author: rharmon112@gmail.com

Abstract

Introduction: The Accreditation Council for Graduate Medical Education (ACGME) requires emergency medicine (EM) residency training programs to monitor residents' progress using standardized milestones. The first assessment of PGY 1 resident milestones occurs midway through the first year and could miss initial deficiencies. Early assessment of PGY 1 EM resident milestones has potential to identify at-risk residents prior to standard midyear evaluations. We developed an orientation syllabus for PGY 1 residents followed by a milestone assessment. Assessment scores helped predict future milestone scores and American Board of Emergency Medicine (ABEM) In-Training Examination (ITE) scores for PGY 1 residents. **Methods:** From 2013 to 2020, we developed and implemented Milestone Evaluation Day (MED), a simulation-based day and written exam assessing PGY 1 EM residents during their first month on the 23 ACGME 1.0 milestones. MED stations included a history and physical with verbal presentation, patient simulation, vascular access, wound management, and airway management. MED, Clinical Competency Committee-generated (CCC-generated) milestone, and ABEM ITE scores were averaged and compared utilizing Pearson's correlation coefficient. **Results:** Of 112 PGY 1 EM residents, 110 (98%) were analyzed over an 8-year period. We observed a moderate positive correlation of MED and CCC-generated milestone scores ($r = .34$, $p < .001$). There was a nonstatistically significant weak positive correlation of MED and ABEM ITE scores ($r = .13$, $p = .17$). **Discussion:** An early assessment of EM milestones in the PGY 1 year can assist in the prediction of CCC-generated milestone scores for PGY 1 residents.

Keywords
Milestones, Assessment, Clinical Skills Assessment/OSCEs, Clinical/Procedural Skills Training, Competency-Based Medical Education (Competencies, Milestones, EPAs), Emergency Medicine, Simulation

Educational Objectives

By using this assessment, facilitators will be able to:

1. Implement Milestone Evaluation Day to assess PGY 1 emergency medicine residents during their first month of residency.
2. Create baseline Accreditation Council for Graduate Medical Education milestone scores for early first-year emergency medicine residents.
3. Predict later Clinical Competency Committee-generated milestone scores.

Citation:
Versalle RL, Todd BR, Chen NW, Turner-Lawrence DE. Early emergency medicine milestone assessment for predicting first-year resident performance. *MedEdPORTAL*. 2024;20:11386.
https://doi.org/10.15766/mep_2374-8265.11386

Introduction

In 2013, the Accreditation Council for Graduate Medical Education (ACGME) outlined 23 emergency medicine (EM) milestones that are used as outcome measures for EM residents as they progress through residency.[1] For each milestone, residents are graded on a proficiency level, with level 1 equating to a medical school graduate and level 4 equating to a graduating EM resident. EM programs utilize a Clinical Competency Committee (CCC) semiannually to assess each resident's current milestones.

Many PGY 1s report no guidance on milestones upon entering residency, creating the potential for underprepared residents.[2] A significant number of residents have critical deficiencies at the beginning of their training, with many PGY 1s not being able to achieve level 1 on several milestones.[3,4] There may be a gap between the self-evaluation and the actual performance of PGY 1s in EM milestones, as they tend to rate themselves higher than their objective assessment.[5] PGY 1s who have

Copyright © 2024 Versalle et al. This is an open-access publication distributed under the terms of the Creative Commons Attribution license.

had milestone-based simulations early in their training report improved confidence and knowledge; however, whether this correlates with improved later CCC-generated milestone scores is unknown.[6]

Currently, there is no standardized process to evaluate the milestones of incoming PGY 1s. This presents a potential missed opportunity to identify residents with initial deficiencies who are at risk for later milestone deficiencies or a poor performance on the American Board of Emergency Medicine (ABEM) In-Training Examination (ITE). Utilizing the theoretical construct of ACGME milestones as a useful measure of resident proficiency, we aimed to develop a summative milestone assessment for PGY 1 residents in their first month of residency.

Methods

Development

The ACGME milestones were designed for the purpose of monitoring resident proficiency throughout training. We developed Milestone Evaluation Day (MED), a 1-day, simulation-based assessment that also included a written exam, which was to be completed at the end of the first month of residency training. During MED, PGY 1s participated in seven timed stations that assessed each resident on skills related to the 23 ACGME 1.0 milestones. The MED assessed level 1 proficiency on all 23 milestones through the following stations: history and physical with patient presentation, patient simulation, intravenous peripheral vascular access, wound management, arterial puncture, airway, and written exam. Station descriptions in Appendix A highlight which milestones were assessed by each station.

Prior to MED, trainees participated in an orientation month that included didactics and hands-on labs correlating with ACGME level 1 milestones. Trainees were made aware of the MED assessment at the beginning of their orientation month and were provided with an introduction to the format of MED approximately 2 weeks prior to their assessment. During the introduction to MED, they were given examples and expectations of level 1 milestone objectives that would be evaluated. Appendix B features an example of our PGY 1 orientation didactic syllabus for the month.

This assessment was created at a 3-year EM residency program with a total of 42 residents based at a tertiary care hospital in southeast Michigan and offered in July of each year.

Equipment/Environment

MED was conducted at our hospital's simulation center, which accommodated the seven stations. MED required specific equipment unique to each station. Lists of specific equipment required for each station are presented in Appendix A.

Personnel

Approximately 10 instructors were required to implement MED typically over a 4-hour time span. A majority of these instructors were emergency department faculty, with one being a senior resident who role-played the patient in station 1A. We enlisted experienced faculty, aiming for consistency by retaining the same faculty at each station annually to minimize interrater variability in scoring and subsequent feedback. The specific personnel required for each station and their roles are outlined in Appendix A. Each evaluator received written instructions for their role before the start of MED.

Implementation

Station 1—history and physical with patient presentation: Station 1 was conducted in faculty offices and consisted of two parts. For part 1A, trainees were provided with a clinical vignette of a patient with symptomatic cholelithiasis and instructed to perform a full history and physical on this patient. The patient was played by a senior resident who had been provided with patient information prior to the simulation. Trainees were given 5 minutes to complete the history and physical. The senior resident assessed trainees on part 1A utilizing the checklist (Appendix C). Trainees were then instructed to go to a different room for part 1B, where a faculty member was present to act as an attending. Trainees were instructed to present the patient to the attending, including differential diagnoses and plans for the patient. Trainees had 5 minutes for patient presentation. The faculty member assessed trainees on part 1B utilizing the checklist (Appendix C). Materials used for station 1, including trainee instructions, evaluator instructions, clinical vignette, and patient information, are provided in Appendix D.

Station 2—patient simulation: Station 2 was conducted in the simulation center utilizing a SimMan (Laerdal). The patient scenario was a case of supraventricular tachycardia (SVT). Trainees were provided with the clinical vignette and instructed to take a focused history and physical as if they were in the emergency department and to carry out diagnosis, treatment, and disposition. A faculty member voiced the SimMan, and a second faculty member played the nurse. The simulated patient was in SVT, and trainees were expected to recognize the dysrhythmia and treat appropriately. Trainees were given 10 minutes for the simulation. A third faculty member was present to observe the simulation and assess trainees utilizing the checklist (Appendix C). Materials used for station 2, including trainee instructions,

Copyright © 2024 Versalle et al. This is an open-access publication distributed under the terms of the Creative Commons Attribution license.

evaluator instructions, clinical vignette, scenario branch points, patient information, and workup results, are provided in Appendix E.

Station 3—venous vascular access: Station 3 was conducted in the simulation center. Trainees were provided with instructions to place a peripheral IV on a venous vascular access trainer. Trainees had 5 minutes for this task. A faculty member was present to run the station and assess the trainees utilizing the checklist (Appendix C). Materials used for station 3, including trainee instructions and evaluator instructions, are provided in Appendix F.

Station 4—wound management: Station 4 was conducted in the simulation center. Trainees were provided with instructions to sterilize, inject local anesthetic, and place three simple interrupted sutures in a 4-cm laceration in a pork belly. Trainees had 10 minutes for this task. A faculty member was present to run the station and assess the trainees utilizing the checklist (Appendix C). Materials used for station 4, including trainee instructions and evaluator instructions, are provided in Appendix G.

Station 5—arterial puncture: Station 5 was conducted in the simulation center. Trainees were provided with instructions to puncture the radial artery on an arterial vascular access trainer. Trainees were given 5 minutes for this task. A faculty member was present to run the station and assess the trainees utilizing the checklist (Appendix C). We provide the materials used for station 5, including trainee instructions and evaluator instructions, in Appendix H.

Station 6—airway: Station 6 was conducted in the simulation center. Trainees were provided with instructions for six airway tasks to perform while at this station, including demonstration of a jaw thrust and chin lift, placement of a nasopharyngeal and oral airway, ability to perform bag mask ventilation/oxygenation, and correct identification of nine upper airway structures. Trainees were provided with a clinical photo of airway anatomy and instructed to identify each structure. Trainees were given 10 minutes at this station. A faculty member was present to run the station and assess the trainees utilizing the checklist (Appendix C). Materials used for station 6, including trainee instructions, evaluator instructions, and airway anatomy with answers, are provided in Appendix I.

Station 7—written exam: Station 7 was conducted in faculty offices from 2013 through 2019. Trainees were provided with a written exam to test their medical knowledge. Evaluators proctored and then graded the exam, giving a final percentage score. Trainees had 30 minutes to take the exam. The exam was modified yearly to reflect content delivered to PGY 1s during their orientation month. The number of questions varied from 20 to 51. Starting in 2020, the written exam station was removed from MED. Instead, residents were instructed to take an online standardized assessment (Rosh Review Intern Assessment, Rosh Review). This online assessment needed to be completed prior to MED, and the final score was used for MED results. Written exam materials for station 7, including trainee instructions, evaluator instructions, and an example of an in-person exam with answer key, are provided in Appendix J.

Feedback
During MED, faculty evaluated PGY 1 residents on specific level 1 actions at each simulation-based station, and individual scores were totaled to create an aggregate score. Aggregate scores for each resident were compiled using station checklists (Appendix C). Due to the time constraints and testing nature of the MED assessment, trainees did not receive score sheets, real-time feedback, or debriefing during MED. Within 1 month of completing the MED, we gave PGY 1 residents a report that summarized their total score for each station and qualitative feedback on their performance based on level 1 milestones. An example of this report is provided in Appendix K.

Validity Evidence
MED was a summative assessment with validity evidence in the area of consequences impacting trainees and faculty. The assessment was constructed to provide a current understanding of the trainees' milestones and thus was not a pass/fail assessment. Residents who performed below ACGME level 1 milestones were provided with feedback and opportunities and resources to remediate their skills. Use of MED and subsequent identification of residents not meeting level 1 milestones allowed for early intervention and targeted improvement.

Assessment
During MED, faculty evaluated PGY 1 residents on specific level 1 actions of ACGME milestones at each simulation-based station. Individual scores were totaled to create an aggregate score. Checklists with level 1 milestones assessed at each station are detailed in Appendix C.

Data Analysis
We conducted a retrospective analysis of MEDs implemented at our residency program from 2013 through 2020. We screened all PGY 1 residents who participated in MED over an 8-year period for inclusion in this analysis. Those who did not complete all

Copyright © 2024 Versalle et al. This is an open-access publication distributed under the terms of the Creative Commons Attribution license.

MED stations were excluded. This analysis was approved by the Oakland University Institutional Review Board (IRB #2018-215, approved September 29, 2021).

EM residents were deidentified prior to data analysis. Cumulative scores for all MED stations were totaled and then averaged for each resident, providing a total MED score. Milestone scores from fall and spring CCC meetings were summed and then averaged, giving a final CCC-generated milestone score. ABEM ITE scores were available in spring of PGY 1 year. Correlations between the PGY 1 MED scores and each PGY 1's cumulative milestone and ABEM ITE scores were assessed utilizing Pearson's correlation coefficient.

Results

We collected data from 110 EM residents out of an available pool of 112 PGY 1 EM residents (98%) over the 8-year period. Of these residents, 39 (35%) were female. We excluded two residents, one who did not attend MED and one who did not have a documented airway score. The mean MED score was 121.3 (*SD* = 10.5, minimum = 91.5, maximum = 142.0). The mean CCC-generated milestone score was 40.6 (*SD* = 3.5, minimum = 29.8, maximum = 54.8). There was a statistically significant positive correlation of MED and CCC-generated milestone scores ($r = .34$, $p < .05$). The mean ABEM ITE score was 71.5 (*SD* = 7.4, minimum = 49.0, maximum = 90.0). There was a nonstatistically significant positive correlation of MED and ABEM ITE scores ($r = .13$, $p = .17$). Score distributions are shown in the Figure.

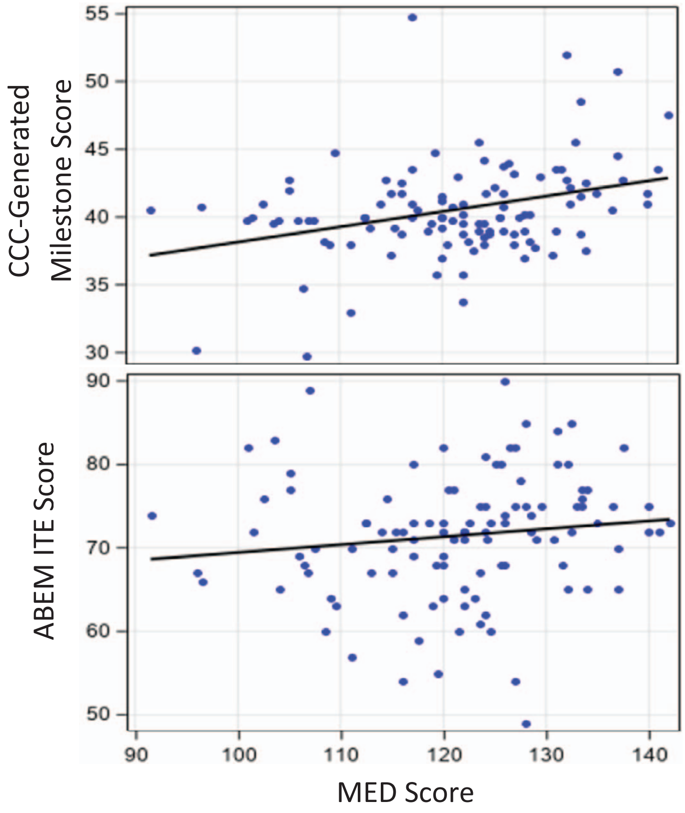

Figure. Score distribution of MED scores compared to ABEM ITE and CCC-generated milestone scores. Abbreviations: ABEM ITE, American Board of Emergency Medicine In-Training Exam; CCC, Clinical Competency Committee; MED, Milestone Evaluation Day.

Further analysis of the data showed that residents scored highest at the wound care station (98%) and lowest at the airway station (83%). Further analysis also revealed that the average in-person written exam score for the first 7 years was 84%. However, for the last year, when the exam was changed to online standardized assessment, the average score dropped to 64%.

Many of the faculty who served as evaluators during MED also served as members of the CCC. Anecdotally, faculty feedback through CCC discussions noted that information from MED was helpful in assigning the PGY 1 milestones. Two of the authors served as CCC chairs and reported the importance of MED in CCC discussions and in identifying residents who might need early intervention to correct for milestone deficiencies.

The effectiveness of MED was evaluated in the context of the consequence area of validity. The ACGME milestone levels mirrored the level of entrustment of residents throughout training. These levels represented a continuum of supervision and autonomy, allowing educators to gauge a learner's progress and competence. ACGME level 1 milestones represented a beginning point for PGY 1 residents. Each station was utilized to assess level 1 milestones and together provide a comprehensive assessment of all 23 ACGME milestones. Performance feedback on MED was provided to residents, so they could have an understanding of their ACGME milestones at the beginning of residency, and to the CCC, to allow faculty to assess current milestones. MED demonstrated effectiveness through its positive correlation with improved CCC milestone scores and, to a lesser extent, ABEM ITE scores, meeting our objectives for the assessment.

Discussion

Residency leadership may find it challenging to identify residents with early milestone deficiencies, emphasizing the importance of conducting an initial assessment of residents early in their training. We implemented MED to generate preliminary PGY 1 milestones and identify residents not meeting them. MED may also help PGY 1 residents more precisely assess their milestones, as historically residents have not been accurate in predicting

Copyright © 2024 Versalle et al. This is an open-access publication distributed under the terms of the Creative Commons Attribution license.

their milestones on self-assessments.[5] While some literature has assessed milestone competencies of early EM trainees and other studies have shown simulation to be useful for assessment of EM milestones,[6-8] none of these have evaluated the prediction of later CCC-generated milestone scores. Our results indicate that an early assessment of ACGME milestones in the PGY 1 year can help predict later CCC-generated milestone scores for PGY 1 residents. This creates an opportunity to identify residents at risk for critical deficiencies early in their training prior to their fall CCC meeting so that improvement initiatives can occur. Early intervention may help residents and programs achieve their expected milestone targets.

We observed a weak positive correlation between MED scores and ABEM ITE scores for PGY 1 residents, although it was not statistically significant. This could be explained in part by the change of exam format in the final year from a written to an online standardized assessment. The online standardized assessment was more difficult than the previously utilized written exam, as it covered a wider range of core content, some of which may not have been explicitly addressed during the July orientation didactics. By identifying PGY 1 residents who struggle with an exam early in their training, programs may be able to initiate early targeted medical knowledge interventions that may result in improved ABEM ITE outcomes. Future studies could investigate if such interventions improve scores on the ABEM qualifying exam.

Our analysis is limited by a small sample size, as MED was conducted at a single trainee site. It was also conducted in a large simulation center with access to simulation materials such as SimMan and vascular access trainers; other programs may not have access to these resources. These limitations may pose a challenge to generalizability; however, we believe MED could be adapted for different residency programs. For example, if a high-fidelity simulator is unavailable, an actor playing the patient would be acceptable. The proctor would need to notify trainees of changes in vitals. Rather than utilizing pork, wound management could be performed on cut fruit, such as bananas or oranges. We utilized faculty offices for many of our stations and believe all the stations could be adapted to this type of space.

Our analysis is also limited by not having collected a pragmatic assessment of MED from our residents. Although we did not formally collect feedback from residents on the MED assessment, they typically expressed a positive view of MED in discussions about their performance. They found it helpful in gaining insight into their current milestone status. Future changes could include creating a written survey evaluating whether second- or third-year residents feel early assessment of milestones and related feedback impacted their study habits or performance. Future studies should evaluate whether tailored remediation based on early milestone assessment is of value to second- or third-year residents.

Finally, our analysis is also limited by the use of ACGME milestones 1.0. Modifications and additions may need to be made to accommodate milestones 2.0. Further directions could include expansion of MED to different EM residency training sites and other specialties utilizing ACGME milestones 2.0. Further studies should assess whether early intervention on lagging milestones can improve future CCC-generated milestone and ABEM ITE scores.

Appendices

A. MED Stations and Schedule.docx
B. Sample EM PGY 1 Orientation Didactic Syllabus.docx
C. MED Checklists.docx
D. MED Station 1 Materials.docx
E. MED Station 2 Materials.docx
F. MED Station 3 Materials.docx
G. MED Station 4 Materials.docx
H. MED Station 5 Materials.docx
I. MED Station 6 Materials.docx
J. MED Station 7 Materials.docx
K. MED Performance Summary.docx

All appendices are peer reviewed as integral parts of the Original Publication.

Rochelle L. Versalle, MD: Third-Year Resident, Department of Emergency Medicine, Oakland University William Beaumont School of Medicine; ORCID: https://orcid.org/0000-0002-7210-7742

Brett R. Todd, MD: Associate Professor, Department of Emergency Medicine, Oakland University William Beaumont School of Medicine

Nai-Wei Chen, PhD: Statistician, Division of Informatics and Biostatistics, Beaumont Institute

Danielle E. Turner-Lawrence, MD: Associate Professor, Department of Emergency Medicine, Oakland University William Beaumont School of Medicine

Disclosures
None to report.

Funding/Support
None to report.

Copyright © 2024 Versalle et al. This is an open-access publication distributed under the terms of the Creative Commons Attribution license.

Ethical Approval

The Oakland University Institutional Review Board approved this project.

References

1. Beeson MS, Holmboe ES, Korte RC, et al. Initial validity analysis of the emergency medicine milestones. *Acad Emerg Med*. 2015;22(7):838-844. https://doi.org/10.1111/acem.12697
2. Santen SA, Rademacher N, Heron SL, Khandelwal S, Hauff S, Hopson L. How competent are emergency medicine interns for level 1 milestones: who is responsible? *Acad Emerg Med*. 2013;20(7):736-739. https://doi.org/10.1111/acem.12162
3. Kinnear B, Bensman R, Held J, O'Toole J, Schauer D, Warm E. Critical deficiency ratings in milestone assessment: a review and case study. *Acad Med*. 2017;92(6):820-826. https://doi.org/10.1097/ACM.0000000000001383
4. Weizberg M, Bond MC, Cassara M, Doty C, Seamon J. Have first-year emergency medicine residents achieved level 1 on care-based milestones? *J Grad Med Educ*. 2015;7(4):589-594. https://doi.org/10.4300/JGME-D-14-00590.1
5. Goldflam K, Bod J, Della-Giustina D, Tsyrulnik A. Emergency medicine residents consistently rate themselves higher than attending assessments on ACGME milestones. *West J Emerg Med*. 2015;16(6):931-935. https://doi.org/10.5811/westjem.2015.8.27247
6. Frallicciardi A, Nowicki T, Herbst M, Flores A. A simulation-based emergency medicine PGY-1 resident orientation curriculum. *MedEdPORTAL*. 2015;11:10115. https://doi.org/10.15766/mep_2374-8265.10115
7. Hauff SR, Hopson LR, Losman E, et al. Programmatic assessment of level 1 milestones in incoming interns. *Acad Emerg Med*. 2014;21(6):694-698. https://doi.org/10.1111/acem.12393
8. Hart D, Bond W, Siegelman JN, et al. Simulation for assessment of milestones in emergency medicine residents. *Acad Emerg Med*. 2018;25(2):205-220. https://doi.org/10.1111/acem.13296

Received: July 6, 2023
Accepted: November 28, 2023
Published: March 12, 2024

Copyright © 2024 Versalle et al. This is an open-access publication distributed under the terms of the Creative Commons Attribution license.
